# Activated SUMOylation restricts MHC class I antigen presentation to confer immune evasion in cancer

**DOI:** 10.1172/JCI152383

**Published:** 2022-05-02

**Authors:** Uta M. Demel, Marlitt Böger, Schayan Yousefian, Corinna Grunert, Le Zhang, Paul W. Hotz, Adrian Gottschlich, Hazal Köse, Konstandina Isaakidis, Dominik Vonficht, Florian Grünschläger, Elena Rohleder, Kristina Wagner, Judith Dönig, Veronika Igl, Bernadette Brzezicha, Francis Baumgartner, Stefan Habringer, Jens Löber, Björn Chapuy, Carl Weidinger, Sebastian Kobold, Simon Haas, Antonia B. Busse, Stefan Müller, Matthias Wirth, Markus Schick, Ulrich Keller

**Affiliations:** 1 Department of Hematology, Oncology and Cancer Immunology, Charité – Universitätsmedizin Berlin, corporate member of Freie Universität Berlin and Humboldt-Universität zu Berlin, Berlin, Germany.; 2 Max-Delbrück-Center for Molecular Medicine, Berlin, Germany.; 3 Clinician Scientist Program, Berlin Institute of Health (BIH), Berlin, Germany.; 4 BIH at Charité – Universitätsmedizin Berlin, Berlin, Germany.; 5 Berlin Institute for Medical Systems Biology, Max Delbrück Center for Molecular Medicine in the Helmholtz Association, Berlin, Germany.; 6 Institute of Biochemistry II, Goethe University Frankfurt, Medical School, Frankfurt, Germany.; 7 Division of Clinical Pharmacology, Department of Medicine IV, Klinikum der Universität München, Munich, Germany.; 8 Heidelberg Institute for Stem Cell Technology and Experimental Medicine (HI-STEM gGmbH), Heidelberg, Germany.; 9 Division of Stem Cells and Cancer, Deutsches Krebsforschungszentrum (DKFZ) and DKFZ-ZMBH Alliance, Heidelberg, Germany.; 10 Faculty of Biosciences, Heidelberg University, Heidelberg, Germany.; 11 Experimental Pharmacology and Oncology Berlin-Buch GmbH (EPO), Berlin, Germany.; 12 Department of Hematology and Oncology, University Medical Center Göttingen, Göttingen, Germany.; 13 Gastroenterology, Infectiology and Rheumatology, Campus Benjamin Franklin, Charité – Universitätsmedizin Berlin, corporate member of Freie Universität Berlin and Humboldt-Universität zu Berlin, Berlin, Germany.; 14 German Center for Translational Cancer Research (DKTK), DKFZ, Heidelberg, Germany.; 15 DKTK, Partner Site Munich, Munich, Germany.; 16 Einheit für Klinische Pharmakologie (EKLiP), Helmholtz Zentrum München, German Research Center for Environmental Health (HMGU), Neuherberg, Germany.

**Keywords:** Immunology, Oncology, Antigen presentation, Immunotherapy, Ubiquitin-proteosome system

## Abstract

Activated SUMOylation is a hallmark of cancer. Starting from a targeted screening for SUMO-regulated immune evasion mechanisms, we identified an evolutionarily conserved function of activated SUMOylation, which attenuated the immunogenicity of tumor cells. Activated SUMOylation allowed cancer cells to evade CD8^+^ T cell–mediated immunosurveillance by suppressing the MHC class I (MHC-I) antigen-processing and presentation machinery (APM). Loss of the MHC-I APM is a frequent cause of resistance to cancer immunotherapies, and the pharmacological inhibition of SUMOylation (SUMOi) resulted in reduced activity of the transcriptional repressor scaffold attachment factor B (SAFB) and induction of the MHC-I APM. Consequently, SUMOi enhanced the presentation of antigens and the susceptibility of tumor cells to CD8^+^ T cell–mediated killing. Importantly, SUMOi also triggered the activation of CD8^+^ T cells and thereby drove a feed-forward loop amplifying the specific antitumor immune response. In summary, we showed that activated SUMOylation allowed tumor cells to evade antitumor immunosurveillance, and we have expanded the understanding of SUMOi as a rational therapeutic strategy for enhancing the efficacy of cancer immunotherapies.

## Introduction

SUMOylation is a posttranslational protein modification that controls the localization, stability, and activity of target proteins (1, 2). SUMOylation has a regulatory function in fundamental cellular processes, such as cell cycle progression, transcription, and chromatin remodeling (3). The process of SUMOylation is highly dynamic and controlled by a well-coordinated enzymatic cascade, including the heterodimeric E1 SUMO-activating enzyme SAE1/UBA2, the E2 SUMO-conjugating enzyme UBC9, and multiple E3 ligases. Mammalian cells express 3 conjugatable SUMO isoforms: SUMO1, SUMO2, and SUMO3. SUMO1 shares about 50% homology with SUMO2 and SUMO3, whereas SUMO2 and SUMO3 are typically referenced as SUMO2/3 given their sequence homology, impeding their discrimination by antibody detection (1, 2). SUMO2 is typically expressed to a much higher extent than SUMO3 in most tissues (2). SUMOylation is fully reversible by SUMO-specific isopeptidases of the SENP family, which deconjugate SUMO from its substrates (4, 5). Of note, dysregulation of oncogenes, such as *MYC*, mutant *KRAS*, or *NOTCH1*, provokes activation of the SUMO pathway and shifts the SUMO equilibrium to a hyperSUMOylation state (1). Although activated SUMOylation is a perceived hallmark of human cancers (1, 6), the mechanistic implications of activated SUMOylation on cancer biology are incompletely understood.

Typically, SUMOylation is activated in response to cellular stress. Even more than their healthy counterparts, cancer cells are subject to various environmental impacts like replicative stress, hypoxia, and specifically, the antitumor immune response of the host immune system (1, 7). Notably, many cancers ultimately evade the immune system by mounting distinct immune evasion strategies (8). Cytotoxic T cells (CTLs) are key players of cellular defense within the adaptive immune response. CTLs recognize foreign antigens processed and presented by the MHC class I (MHC-I) antigen processing and presentation machinery (APM) of target cells (9, 10). Antigen processing includes several subsequent steps, including degradation of cellular proteins by the proteasome and the immunoproteasome, TAP transporter-mediated translocation of processed peptides into the ER, and peptide loading onto the MHC-I–β-2-microglobulin (B2M) complex, followed by a transport to the cell surface and subsequent antigen presentation to CTLs. Thus, the MHC-I–presenting pathway is essential for immune recognition and tumor elimination through CTLs (11).

The sufficient capacity for antigen presentation is also critical for therapies potentiating antitumor immunity, such as immune checkpoint inhibition or bispecific T cell engagers. Despite the striking success of immunotherapies in some entities, other diseases are still considered as primary immune-refractory or exhibit an acquired immunotherapy resistance (12, 13). Not surprisingly, loss or downregulation of the MHC-I APM is a common cause of primary as well as acquired resistance to cancer immunotherapies (14). Strategies overcoming this particular immune escape mechanism have recently become a promising research goal to enhance the efficacy of cancer immunotherapies. Whereas genetic alterations in genes encoding members of the MHC-I/APM or regulatory pathways (e.g., the IFN-γ response pathway) cause irreversible MHC-I defects (15, 16), transcriptional repression of MHC-I genes is also observed in a number of tumor entities (17, 18) and contributes to resistance to cancer immunotherapy (19). Besides genetic alterations in MHC APM components, transcriptional silencing of the MHC-I APM is a potentially reversible mechanism of immune escape that could be exploited therapeutically.

In this study, we aimed to investigate the role of activated SUMOylation as a cancer immune evasion strategy. We have uncovered a conserved role of SUMOylation in transcriptional silencing of the MHC-I/APM pathway and expand the understanding of SUMO inhibition (SUMOi) as a rational therapeutic strategy to improve efficacy of cancer immunotherapies.

## Results

### SUMOi restores the MHC-I antigen processing and presentation pathway.

**Figure 1:**
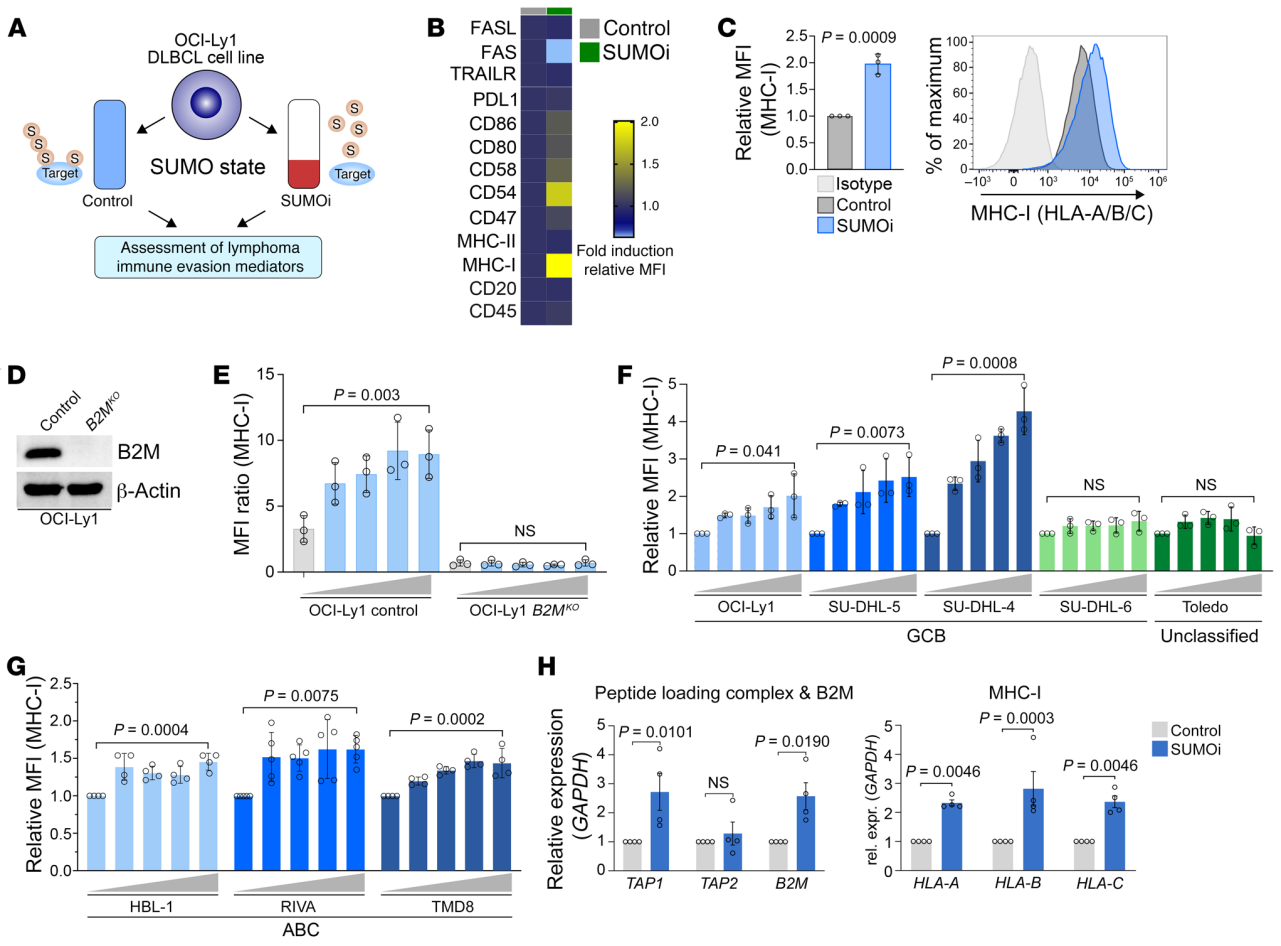
SUMOi induces the MHC-I antigen processing and presentation pathway. (**A**) Outline of the experimental setup for the identification of SUMO-dependent lymphoma immune evasion mechanisms. (**B**) Flow cytometry–based heatmap displaying the fold induction of MFI of immune evasion–associated surface markers of OCI-Ly1 cells treated with 40 nM SUMOi or control for 72 hours (*n =* 3). (**C**) Flow cytometric analysis of MHC-I expression of OCI-Ly1 cells treated with 40 nM SUMOi or DMSO (*n =* 3). Data represent the mean ± SD. *P* value was determined by unpaired *t* test. (**D**) Immunoblot analysis of OCI-Ly1 control and *B2M^KO^* cell lines. (**E**) Flow cytometric analysis of MHC-I expression of OCI-Ly1 control and *B2M^KO^* cell lines treated with increasing SUMOi concentrations (0, 20, 40, 80, 160 nM) for 72 hours (*n =* 3). Data represent the mean ± SD. *P* value was determined by ANOVA with Tukey’s post hoc test. (**F**) Flow cytometric analysis of MHC-I expression on 4 germinal center B cell–like cell lines and 1 unclassified DLBCL cell line treated with increasing concentrations of SUMOi (0, 20, 40, 80, 160 nM) for 72 hours (*n =* 3). Data represent the mean ± SD. *P* values were determined by ANOVA with Tukey’s post hoc test. (**G**) Flow cytometric analysis of MHC-I expression on 3 activated B cell DLBCL cell lines treated with increasing concentrations of SUMOi (RIVA: 0, 20, 40, 80, 160 nM (*n =* 5); HBL-1: 0, 2.5, 5, 10, 20 nM (*n =* 4); TMD8: 0, 5, 10, 20, 40 nM (*n =* 4)) for 72 hours. Data represent the mean ± SD. *P* values were determined by ANOVA with Tukey’s post hoc test. (**H**) mRNA expression analysis of the indicated MHC-I APM genes in SU-DHL-4 cells treated with SUMOi (100 nM, 72 h) or control (*n =* 4). Data represent the mean ± SEM. *P* values were determined by unpaired *t* test.

Among all cancers, B cell non–Hodgkin lymphomas (BCLs) are unique in that the cancer cells themselves are antigen-presenting cells (APCs) (20). Moreover, BCLs are frequently characterized by both activated SUMOylation and distinct immune evasion strategies (20, 21). To systematically assess the role of SUMOylation for lymphoma immune evasion mechanisms, we performed a flow cytometry–based screening in the human diffuse large B cell lymphoma (DLBCL) cell line OCI-Ly1 (Figure 1A) targeting a set of surface molecules with well-known functions for lymphoma immune surveillance (20). To block SUMOylation, we treated OCI-Ly1 cells with the selective SUMOi TAK-981, inhibiting activation of SUMO by the E1 enzyme UBA2 (22). SUMOi dramatically reduced the level of SUMO-modified proteins in DLBCL cell lines (Supplemental Figure 1A). SUMOi-treated cells showed a striking induction of MHC-I expression, whereas the expression of all other analyzed immune evasion proteins was not or only slightly affected (Figure 1, B and C). MHC-I holds a key role in tumor immune recognition, and tumors frequently subvert MHC-I peptide presentation to evade CD8^+^ T cell recognition. Of note, loss of the MHC-I/APM pathway is an established mechanism for acquired resistance to immune checkpoint blockade (ICB) (23) and is frequently absent in DLBCL (24, 25). B2M is a central player of the MHC-I APM, and *B2M* is inactivated in up to one-third of patients with DLBCL (24). *B2M* knockout in OCI-Ly1 cells led to a dramatic reduction of MHC-I expression (Figure 1, D and E). Whereas SUMOi treatment increased MHC-I expression on OCI-Ly1 control cells, SUMOi treatment did not affect MHC-I expression on OCI-Ly1 *B2M^KO^* cells (Figure 1E). However, a substantial fraction of human primary DLBCLs does not harbor genetic alterations of the MHC-I/APM pathway, revealing the potential for therapeutic induction of the MHC-I APM. To substantiate our finding in a larger informative DLBCL cell line panel, we analyzed the effects of SUMOi on MHC-I expression on OCI-Ly1, SU-DHL-4, SU-DHL-5, SU-DHL-6, and Toledo cells. SUMOi treatment induced MHC-I expression in a dose-dependent manner in OCI-Ly1, SU-DHL-4, and SU-DHL-5 cells (Figure 1F) but did not affect MHC-I expression on SU-DHL-6 and Toledo cells (Figure 1F). Of note, SUMOi also restored MHC-I expression in the activated B cell subtype DLBCL cell lines HBL-1, RIVA, and TMD8 (Figure 1G) and in murine B cell lymphoma cell lines (Supplemental Figure 1B), revealing a highly conserved function of SUMOylation in repressing MHC-I expression.

MHC-I processing and peptide presentation are regulated by a complex network of multiple cellular proteins (26). To investigate whether SUMOylation transcriptionally suppresses critical genes encoding the MHC-I APM, we performed qPCR analysis of SUMOi-treated SU-DHL-4 and OCI-Ly1 cells. Intriguingly, in comparison to control cells, inhibition of SUMOylation induced expression of multiple APM genes, including those encoding immunoproteasome components (*LMP2*, *LMP7*), peptide transporters associated with antigen processing (*TAP1*), and components of the MHC-I class molecule (*HLA-A*, *HLA-B*, *HLA-C*, and *B2M*) (Figure 1H and Supplemental Figure 1, C and D; supplemental material available online with this article; https://doi.org/10.1172/JCI152383DS1). To explore the functional effect of SUMOi treatment on antigen processing and presentation in a model system with endogenous antigen expression, we treated B16-OVA cells with SUMOi and quantified cell surface MHC-I (H-2Kb) and OVA peptide bound MHC-I (SIINFEKL:Kb). SUMOi treatment increased SIINFEKL:Kb, revealing that SUMOi also affected antigen processing (Supplemental Figure 1, E and F).

**Figure 2:**
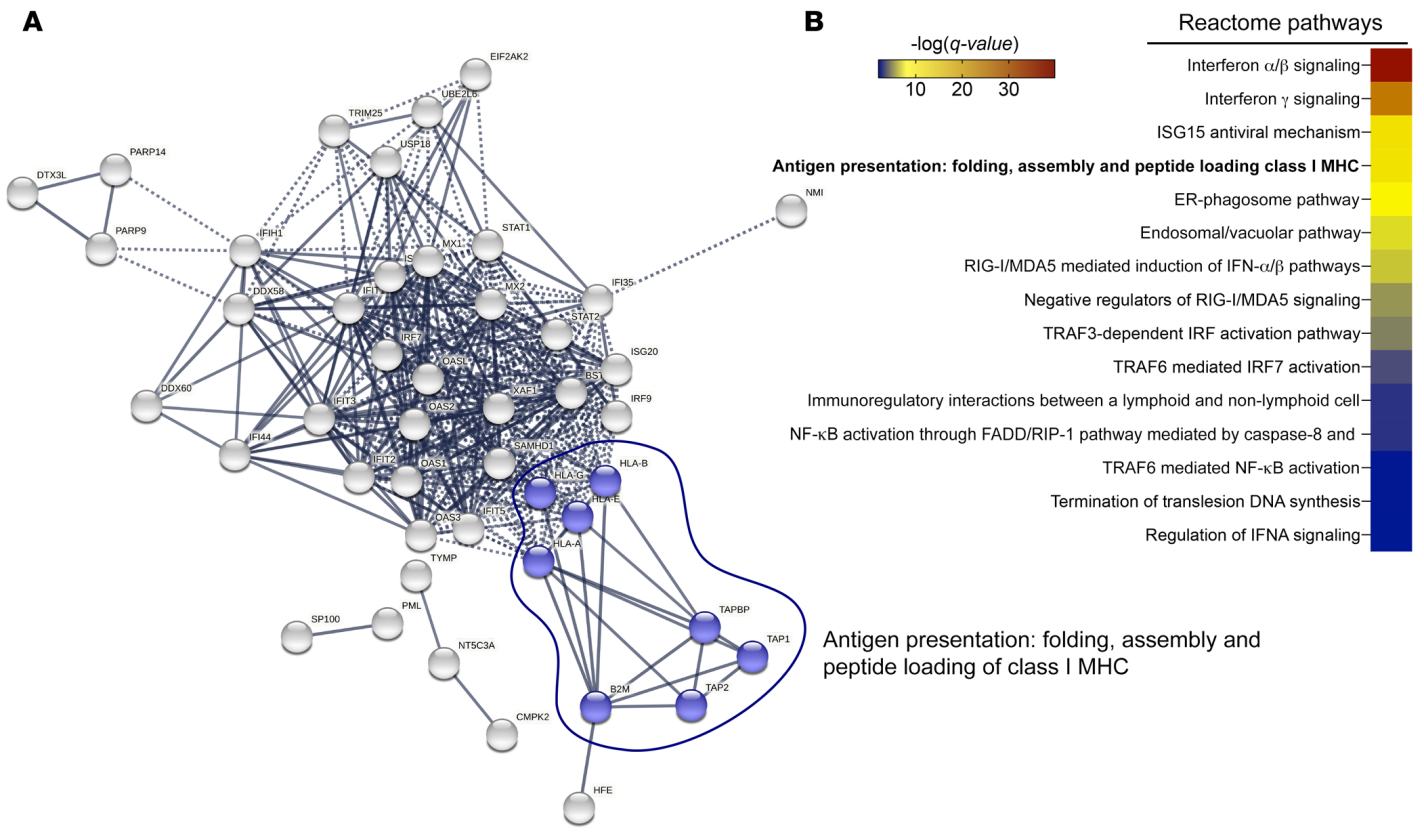
MS-based proteome analysis of SUMOi-treated DLBCL cell lines. (**A**) STRING network analysis depicting the interconnection of proteins enriched after SUMOi treatment of human OCI-Ly1 and SU-DHL-4 DLBCL cell lines. Proteins enriched in OCI-Ly1, SU-DHL-4, or both cell lines were used as input for the network analysis. Only connected proteins are shown. (**B**) Proteins enriched in OCI-Ly1, SU-DHL-4, or both cell lines were analyzed using the Reactome database. The color-coded FDR *q* value is shown for all significantly enriched pathways.

To comprehensively validate our findings on a global and unbiased level, we performed mass spectrometry–based (MS-based) proteome analysis of SUMOi-treated OCI-Ly1 and SU-DHL-4 DLBCL cell lines. Whereas the effects of SUMOi on the overall proteome were moderate, we detected an induction of the MHC-I/APM pathway members HLA-A, HLA-B, B2M, TAP1, TAP1, and TAPBP (Figure 2A, Supplemental Figure 2, and Supplemental Tables 1 and 2). This was further consolidated by pathway analysis showing a remarkable enrichment of the pathway “Antigen Presentation: Folding, assembly and peptide loading of class I MHC” (Figure 2B). Of note, this was fully in line with transcriptome profiling of SUMOi-treated SU-DHL-4 cells (Supplemental Figure 3).

In summary, we identified a conserved role of activated SUMOylation in restricting the MHC-I/APM pathway and showed that inhibition of SUMOylation restored the MHC-I/APM pathway in B cell lymphoma (BCL) cells.

### MYC-induced suppression of the MHC-I/APM pathway is dependent on SUMOylation and confers immune evasion.

**Figure 3:**
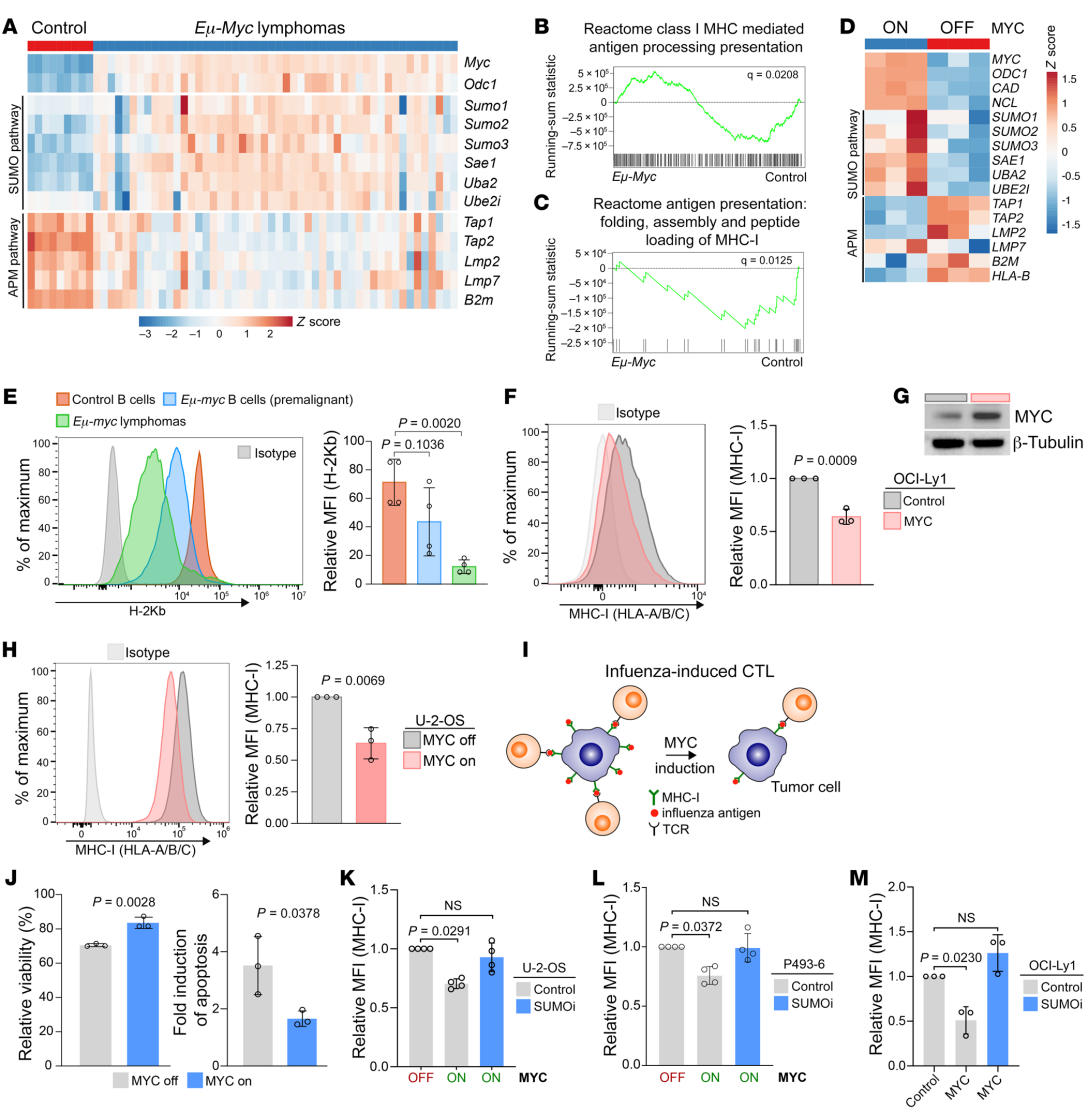
MYC-driven suppression of the MHC-I/APM pathway confers immune evasion and can be restored by SUMOi. (**A**–**C**) Expression of the indicated genes in murine *Eμ-Myc* lymphomas compared with that in control B cells in the GSE7897 data set in the NCBI’s GEO and GSEA analysis. (**D**) Expression of the indicated genes after repression of MYC for 24 hours in the human P493-6 cell line in the GSE32219 data set in the NCBI’s GEO. (**E**) Surface MHC-I expression on B cells derived from WT mice (*n =* 4), premalignant *Eμ-myc* mice (*n =* 4), and *Eμ-myc* lymphomas (*n =* 4). Data represent the mean ± SD. *P* values were determined by ANOVA with Tukey’s post hoc test. (**F**) MHC-I expression of OCI-Ly1 control and MYC cell lines. Data represent the mean ± SD. *P* value was determined by unpaired *t* test. (**G**) Immunoblot analysis of OCI-Ly1 control and MYC cell lines. (**H**) MHC-I expression of U-2-OS cells after induction of MYC for 48 hours. Data represent the mean ± SD. *P* value was determined by unpaired *t* test. (**I**) Experimental setup of the coculture assay performed to assess CTL-mediated cytolysis. (**J**) Flow cytometric analysis of cell death and apoptosis of U-2-OS cells following MYC induction (48 h), after incubation for 4.5 hours with CTLs at an effector/target ratio of 5:1. Viability was determined by DAPI and annexin V staining (*n =* 3). Data represent the mean ± SD. *P* values were determined by unpaired *t* test. (**K**) MHC-I expression of U-2-OS cells treated with SUMOi (100 nM, 72 h) and after induction of MYC for 48 hours (*n =* 4). Data represent the mean ± SD. Statistical significance was determined by ANOVA with Tukey’s post hoc test. (**L**) MHC I expression on P493-6 cells treated with SUMOi (60 nM, 48 h) and repression of MYC for 48 hours (*n =* 3). Data represent the mean ± SD. Statistical significance was determined by ANOVA with Tukey’s post hoc test. (**M**) MHC-I expression on the OCI-Ly1 cells described in **G** treated with SUMOi (100 nM, 72 h) (*n =* 3). Data represent the mean ± SD. Statistical significance was determined by ANOVA with Tukey’s post hoc test.

Activation of MYC is a common feature in DLBCL and causes enhanced protein SUMOylation (21, 27, 28). Increased MYC expression has been associated with suppression of the MHC-I pathway in studies from more than 20 years ago (29). However, the functional consequences of SUMOylation in the context of activated MYC signaling regarding the antitumor immune response have remained elusive. To investigate the association of activated SUMOylation triggered by MYC activation and suppression of antigen presentation in B cell lymphomagenesis, we analyzed a data set of mRNA expression in the NCBI’s Gene Expression Omnibus (GEO) database (GEO GSE7897) comparing WT B cells with MYC-driven BCLs derived from *Eμ-myc* mice. Activated MYC signaling was associated with an increase in SUMO pathway expression and at the same time with a suppression of the APM pathway (Figure 3A). Moreover, GSEA analysis showed depletion of the “Antigen processing and presentation pathway” in murine MYC-driven BCLs (Figure 3, B and C). Additionally, we analyzed the effects of MYC in a mRNA data set of the human P493-6 B cell line carrying a tetracycline-repressible *MYC* transgene (30). Consistently, MYC repression caused suppression of the SUMO pathway and oppositely induction of the antigen presentation pathway (Figure 3D). To confirm these findings in an in vivo lymphomagenesis model, we analyzed the impact of oncogenic MYC on MHC-I levels by comparing *Eμ-myc* lymphomas, premalignant *Eμ-myc* B cells, and WT B cells. Remarkably, MHC-I surface expression was gradually repressed in the course of lymphomagenesis (Figure 3E), which is characterized by gradually increasing MYC levels (22). Moreover, ectopic expression of MYC in the human OCI-Ly1 DLBCL cell lines reduced MHC-I surface expression (Figure 3, F and G). To investigate whether MYC-induced suppression of MHC-I is a conserved mechanisms across tumor entities, we also applied the human sarcoma cell line U-2-OS with doxycycline-inducible MYC (31). Notably, the induction of MYC directly reduced MHC-I surface expression (Figure 3H), revealing a conserved mechanism.

To explore the functional consequences of MYC-induced repression of MHC-I in tumor cells, we established a coculture model system. U-2-OS cells bearing a conditional *MYC* gene (31) were loaded with an influenza peptide before coculturing with CTLs that specifically recognize the MHC-I bound influenza peptide (Figure 3I). U-2-OS cells with lower MHC-I due to MYC activation were less sensitive to antigen-specific T cell killing, indicated by higher viability and lower rate of apoptosis (Figure 3J). To test whether the monitored effects are due to the reduced MHC-I expression, we used siRNA targeting of the *HLA-A* gene to specifically deplete MHC-I. The reduction of MHC-I (Supplemental Figure 4A) and the effect on viability and apoptosis induced by CTLs (Supplemental Figure 4B) were comparable to the effect after MYC-induced suppression of MHC-I, pointing to a direct functional consequence of MYC activation on evasion of CD8^+^ T cell immunosurveillance. Finally, to investigate whether MYC-induced suppression of MHC-I can be restored by inhibition of SUMOylation, we tested the effect of SUMOi in U-2-OS, P493-6, and OCI-Ly1 cells. Remarkably, MYC-induced suppression of MHC-I was fully restored by SUMOi treatment in all 3 cell lines (Figure 3, K–M).

In summary, these data showed that MYC-induced suppression of MHC-I conferred evasion to CD8^+^ T cell immunosurveillance and identified a regulatory mechanism of MHC-I abundance and function, which can be restored by SUMOi.

### Activated SUMOylation is associated with tumor-infiltrating T cells.

**Figure 4:**
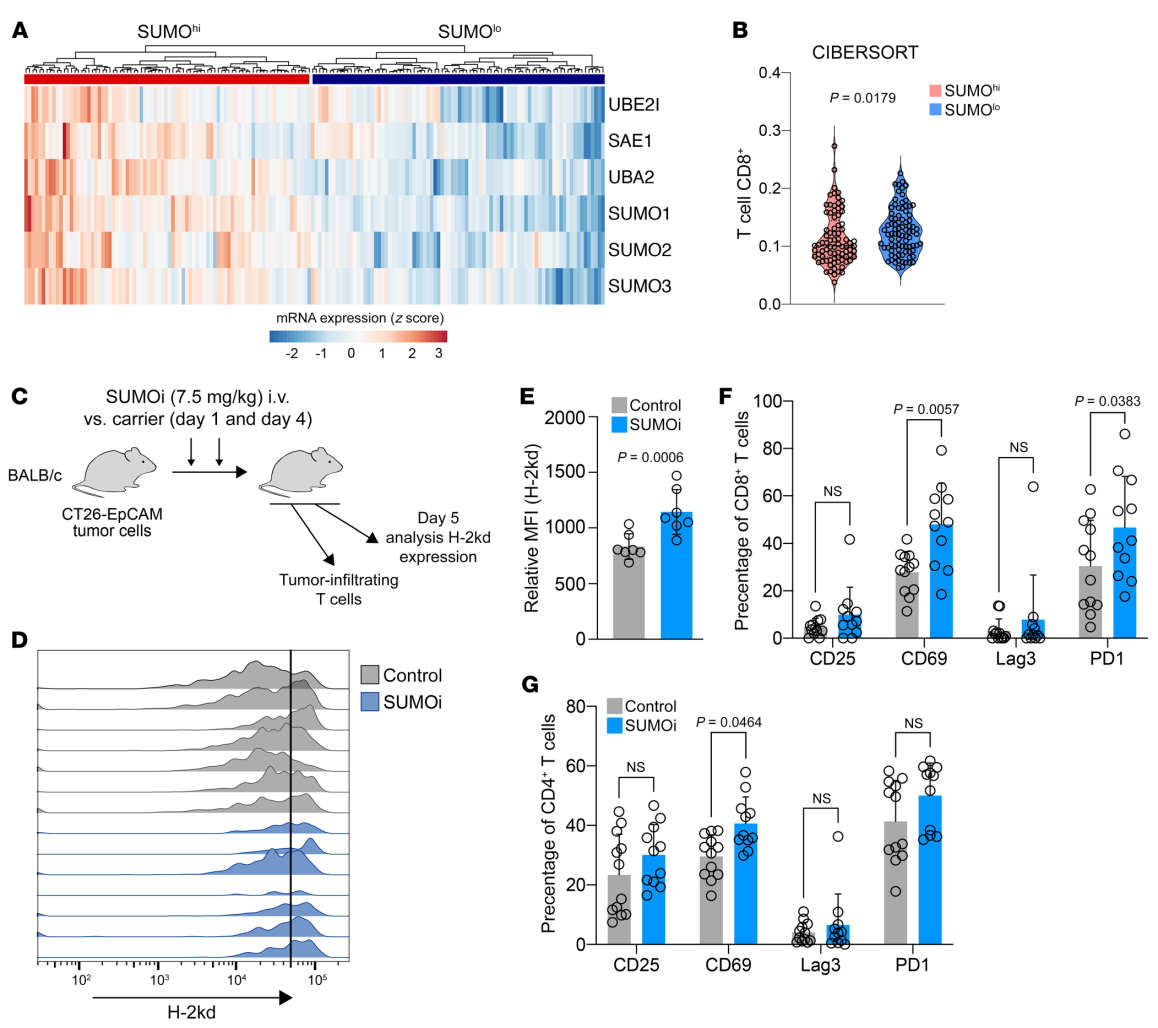
Activated SUMOylation is associated with tumor-infiltrating T cells. (**A**) Expression of SUMO core machinery genes in primary DLBCL samples (*n =* 176) in the GSE4475 data set in NCBI’s GEO clustering in a SUMO^hi^ and SUMO^lo^ cell population. (**B**) Analysis of tumor-infiltrating CD8^+^ T cells with CIBERSORT (68) in the SUMO^hi^ and SUMO^lo^ cell populations described in **A**. *P* value was determined by Mann-Whitney *U* test. (**C**) Experimental workflow for the analysis of H-2kd expression and tumor-infiltrating T cells in CT-26 tumors from mice treated with either SUMOi or carrier control. (**D**) H-2kd expression of murine CT26-EpCAM tumor cells on day 5 after SUMOi or carrier treatment on day 1 and day 4. (**E**) Quantification of H-2kd expression in murine CT26-EpCAM tumor cells on day 5 after SUMOi (*n =* 7) or carrier (*n =* 7) treatment on day 1 and day 4. Data represent the mean ± SD. *P* value was determined by unpaired *t* test. (**F** and **G**) Flow cytometry–based analysis of tumor-infiltrating CD8^+^ and CD4^+^ T cells with the indicated surface markers. Control, *n =* 12; SUMOi, *n =* 11. Data represent the mean ± SD. *P* values were determined by ANOVA with Šidák’s correction.

The MHC-I pathway is critical for the efficacy of ICB, and earlier studies have identified enriched protein expression of the MHC-I/APM pathway members in patients with melanoma responding to anti-PD1 therapy (Supplemental Figure 4C and ref. 32), a standard therapy for patients with melanoma (32, 33). Therefore, we analyzed the effects of MYC in published data sets of this entity. To test the association of MYC, SUMOylation, and the MHC-I pathway, we compared primary tumor samples with *MYC* amplification against samples without *MYC* amplification. Whereas *MYC* and *UBA2*, one of the rate-limiting factors of the SUMO conjugation machinery, were induced in the *MYC* amplified cohort, the expression of the genes encoding the MHC-I complex were reduced (Supplemental Figure 4D), which is in line with our experimental findings of MYC as positive regulator of SUMOylation in BCLs (Figure 3, A–D) and negative regulator of the MHC-I APM (Figure 3, E–H). Moreover, *MYC* and *UBA2* expression was inversely correlated with the presence of CD8^+^ T cells in patients with melanoma (Supplemental Figure 4E), which is a predictive biomarker for the success of ICB (34). To test this association in patients with DLBCL, we analyzed the expression of the SUMO core machinery in primary DLBCL patient samples (35) and identified a SUMO^hi^ and a SUMO^lo^ population (Figure 4A). As expected, MYC signaling was heavily enriched in the SUMO^hi^ population (Supplemental Figure 5, A and B). Of note, the fraction of tumor-infiltrating CD8^+^ T cells was significantly lower in the SUMO^hi^ subgroup (Figure 4B). To consolidate these findings in an established and highly immunogenic in vivo tumor model (36, 37), we treated mice bearing syngeneic CT-26 tumor cells with SUMOi or carrier control to generate direct experimental evidence for the effect of SUMOi for T cell infiltration (Figure 4C). SUMOi treatment led to a significant induction of MHC-I (H-2kd) expression on CT-26 tumor cells (Figure 4, D and E), which was associated with an activation of tumor-infiltrating T cells (Figure 4, F and G).

In summary, these data revealed that activated SUMOylation in tumor cells was associated with the abundance and lower activity of tumor-infiltrating T cells, which can be enhanced by SUMOi.

### Activated SUMOylation restricts cytokine-dependent induction of MHC-I.

**Figure 5:**
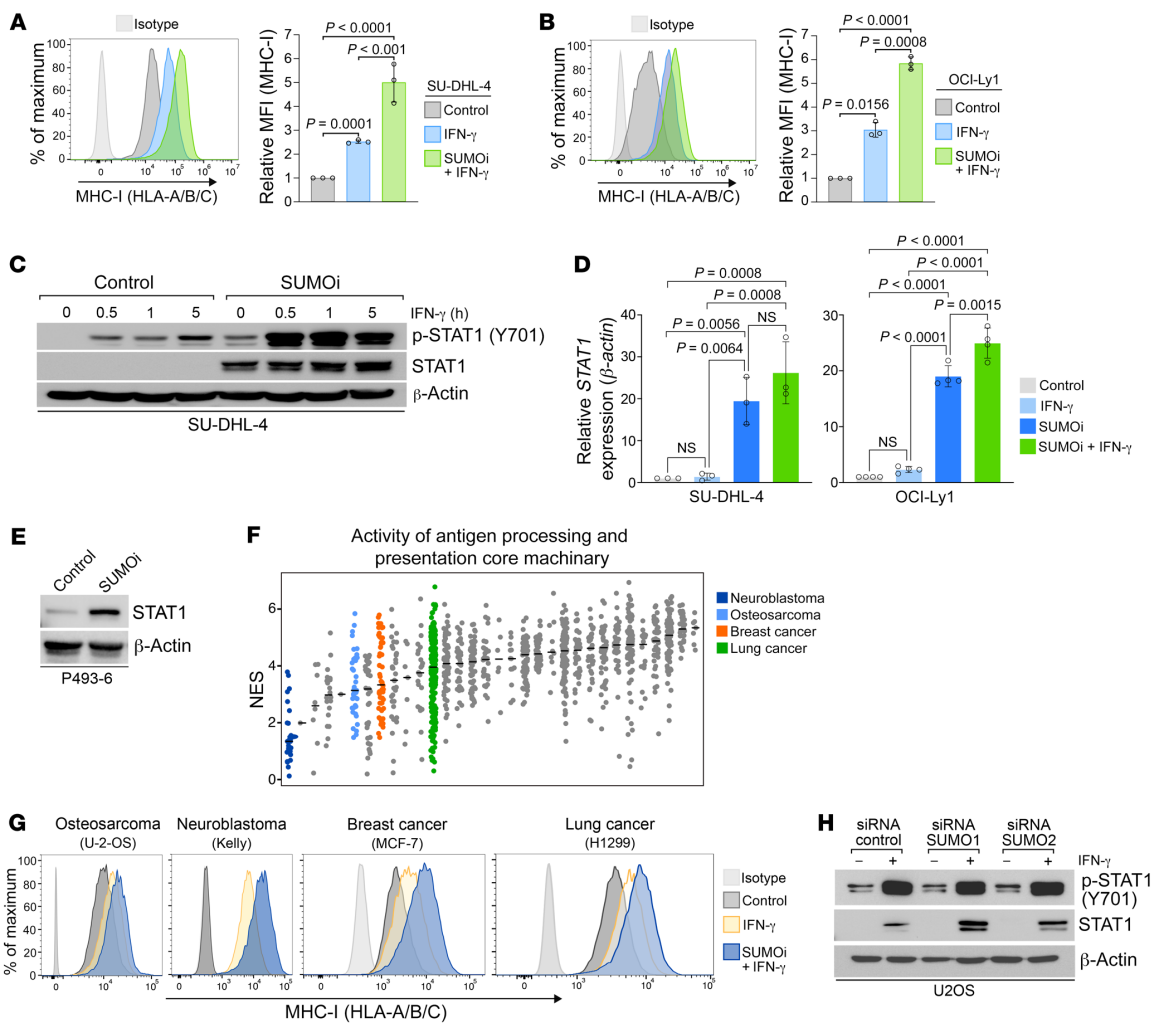
Activated SUMOylation restricts cytokine-dependent induction of MHC-I. (**A**) MHC-I expression on SU-DHL-4 cells treated either with control, SUMOi (100 nM, 72 h), or SUMOi (100 nM, 72 h) and IFN-γ (100 U/mL) for 24 hours (*n =* 3). Data represent the mean ± SD. *P* values were determined by ANOVA with Tukey’s post hoc test. (**B**) MHC-I expression of OCI-Ly1 cells treated with control, SUMOi (40 nM, 72 h), or SUMOi (40 nM, 72 h) and IFN-γ (100 U/mL) for 24 hours (*n =* 3). Data represent the mean ± SD. *P* values were determined by ANOVA with Tukey’s post hoc test. (**C**) Immunoblot analysis of SU-DHL-4 cells treated with IFN-γ (100 U/mL) for the indicated durations after pretreatment with SUMOi (100 nM) or control for 72 hours. (**D**) *STAT1* mRNA expression analysis of SU-DHL-4 (100 nM, 72 h; *n =* 3) and OCI-Ly1 (40 nM, 72 h; *n =* 4) cells treated with control or SUMOi and IFN-γ (100 U/mL) for 1 hour, as indicated. Data represent the mean ± SD. *P* values were determined by ANOVA with Tukey’s post hoc test. (**E**) Immunoblot analysis of P493-6 cells treated with 60 nM SUMOi for 48 hours. (**F**) Activity of the antigen processing and presentation core machinery determined with ssGSEA in cell lines listed in the Cancer Cell Line Encyclopedia. Each dot represents an individual cancer cell line (NES). Horizontal black lines indicate the median. (**G**) MHC-I expression after incubation with IFN-γ (100 U/mL) for 24 hours in the indicated cell lines pretreated with either SUMOi (U-2-OS, Kelly, MCF-7: 100 nM; H1299: 500 nM) or control for 72 hours. (**H**) Immunoblot analysis of U-2-OS cells after transfection with specific SUMO1, SUMO2, or control siRNAs (72 h) and treatment or not with IFN-γ (100 U/mL) for 24 hours.

Driven by the finding that MYC could suppress MHC-I via the activity of the SUMO pathway, we aimed to unravel the molecular mechanism. In response to cytokines like IFN-γ, the MHC-I APM is tightly coordinated by the activity of STAT1 (26). Importantly, MYC has been described as a negative regulator of STAT1 activity (38) and SUMOylation is a critical player in repression of cytokine signaling in the immune response (39). Although SUMOylation of STAT1 limits its phosphorylation and the cellular responsiveness to IFN-γ (40), SUMOi substantially amplified IFN-γ–induced MHC-I induction in DLBCL cell lines (Figure 5, A and B) without affecting the surface expression of the IFN-γ receptor, IFN-γRα (Supplemental Figure 6A). This prompted us to investigate whether SUMOi directly affects STAT1 phosphorylation. Inhibition of SUMOylation amplified IFN-γ–induced STAT1 phosphorylation (Figure 5C and Supplemental Figure 6B) and directly increased STAT1 protein and transcript abundance (Figure 2A, Figure 5, C–E, and Supplemental Figure 6B), thereby priming the STAT1 pathway for IFN-γ signaling. Importantly, SUMOi treatment also caused STAT1 phosphorylation in the absence of IFN-γ. These data thus revealed the presence of additional SUMO-mediated mechanisms affecting the activity of the STAT1 pathway.

Next, we asked whether the SUMOylation-based restriction of IFN-γ–induced MHC-I expression is a general mechanism in cancer. To this end, we applied single sample gene set enrichment analysis (ssGSEA) and classified all tumor types represented in the Cancer Cell Line Encyclopedia (CCLE) according to the activity of the antigen processing and presentation core machinery (Figure 5F). Among these, osteosarcoma, neuroblastoma, breast cancer, and lung cancer exhibited remarkably low activity of the APM pathway. Supporting a conserved role of SUMOylation in silencing of the MHC-I/APM pathway, pharmacological inhibition of SUMOylation significantly amplified the IFN-γ–dependent induction of MHC-I levels in osteosarcoma, neuroblastoma, breast cancer, and lung cancer cells (Figure 5G and Supplemental Figure 6C).

**Figure 6:**
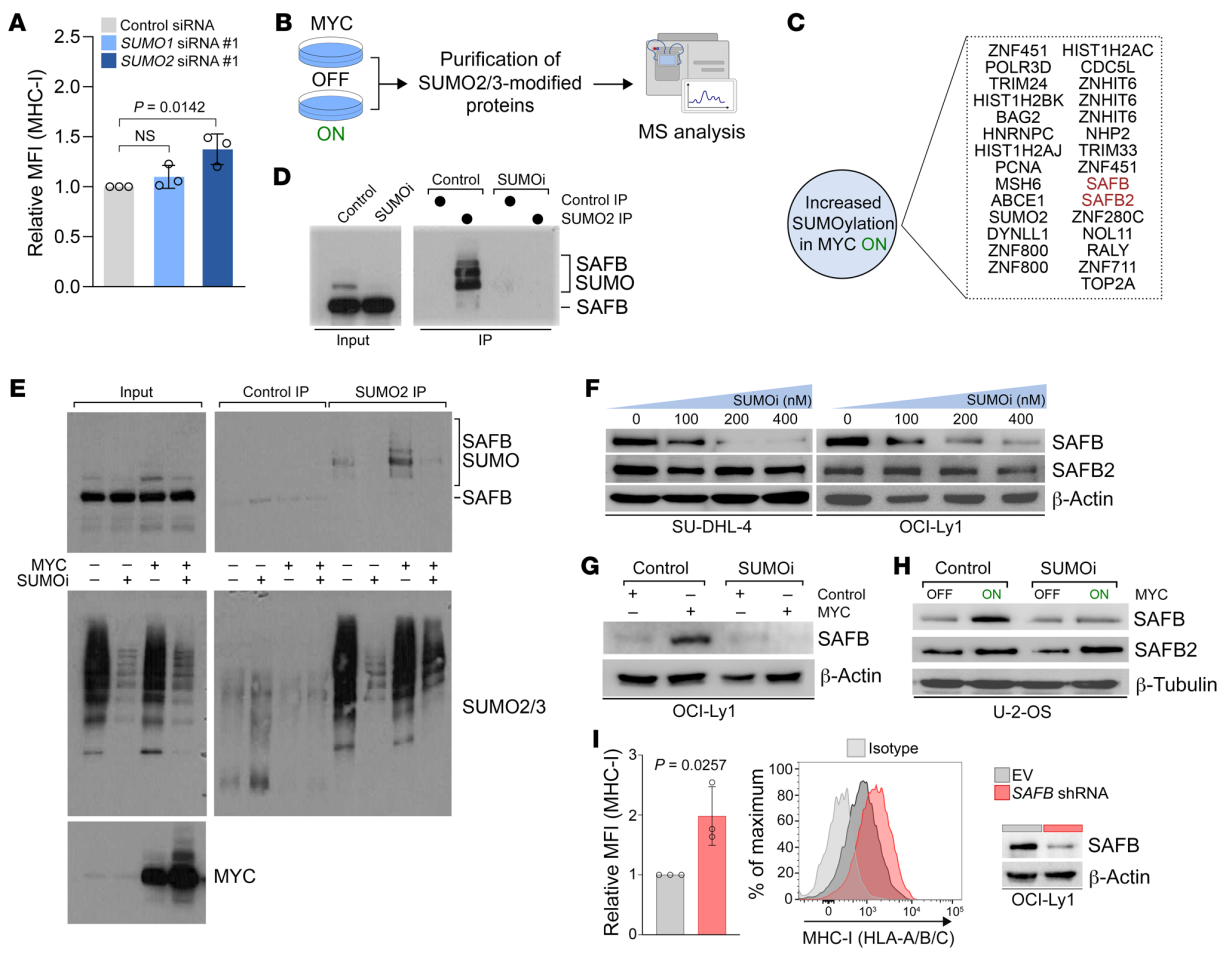
MYC-induced SUMOylation of SAFB suppresses the MHC-I/APM pathway. (**A**) MHC-I expression of U-2-OS cells after transfection with specific SUMO1, SUMO2, or control siRNAs (72 h, *n =* 3). Data represent the mean ± SD. Statistical significance was determined by ANOVA with Tukey’s post hoc test. (**B**) Outline of the experimental setup for the identification of MYC-induced differentially SUMOylated proteins. SUMO2/3-modified proteins were purified from U-2-OS cells after MYC induction for 48 hours and analyzed by MS. (**C**) Schematic illustration summarizing the results of quantitative MS analysis from U-2-OS cells after MYC induction for 48 hours. The experiment was performed in triplicate. (**D**) Immunoblot analysis of U-2-OS cells treated with 100 nM SUMOi or control for 72 hours and IP with either SUMO2 or a control antibody. (**E**) Immunoblot analysis of U-2-OS cells treated with 100 nM SUMOi or control for 72 hours after 48 hours of MYC induction and IP with either SUMO2 or control antibody. (**F**) Immunoblot analysis of SU-DHL-4 and OCI-Ly1 cells treated with the indicated concentrations of SUMOi (0, 100, 200, 400 nM) or control for 72 hours. (**G**) Immunoblot analysis of OCI-Ly1 cells transduced with an MYC expression plasmid or a control plasmid. The cells were treated with either 100 nM SUMOi or control for 72 hours. (**H**) Immunoblot analysis of U-2-OS cells after 48 hours of MYC induction, treated with either 100 nM SUMOi or control for 72 hours. (**I**) MHC-I expression of OCI-Ly1 cells after transduction with a specific *SAFB* shRNA or a control vector (*n =* 3). Data represent the mean ± SD. *P* value was determined by unpaired *t* test. Immunoblot analysis of the respective OCI-Ly1 cells.

To gain further insights into the molecular mechanisms and to explain the general suppression of MHC-I in cancers with activated SUMOylation, we depleted SUMO1 and SUMO2/3 with specific siRNAs. The siRNAs specifically depleted the mRNA expression of SUMO1 and SUMO2 and the levels of SUMO1- and SUMO2/3-modified proteins, respectively (Supplemental Figure 6, D–G). Next, we treated the cells with IFN-γ to activate STAT1 signaling. STAT1 levels were increased upon depletion of SUMO1 as well as SUMO2/3 (Figure 5H and Supplemental Figure 6H). Despite the increase of STAT1 in SUMO1- and SUMO2/3-depleted cells, only depletion of SUMO2/3 induced MHC-I expression (Figure 6A and Supplemental Figure 6I). From these data, we concluded that besides SUMO-dependent restriction of the IFN-γ/STAT1 axis, an additional mechanism must control suppression of MHC-I subsequent to activation of the SUMO pathway.

In summary, we showed that activated SUMOylation restricted the cytokine-dependent induction of MHC-I and identified an additional SUMO-mediated mechanism regulating basal repression of the MHC-I/APM pathway in cancer.

### MYC-induced SUMO2/3 modification of SAFB is linked to MHC-I repression.

To further explore the mechanism of MYC-induced, SUMO2/3-mediated repression of MHC-I (Figure 6A), we aimed to identify the cellular targets of MYC-induced SUMOylation. To this end, we enriched SUMO2/3-modified peptides from LysC-digested cell lysates by anti-SUMO2 IP and identified SUMO2/3 sites by analyzing further AspN digested peptides using a label-free MS approach (Figure 6B and ref. 41). When comparing SUMO targets of MYC-induced cells with control cells, 42 SUMO2/3 sites in 34 proteins were exclusively identified upon MYC induction (Supplemental Table 4). To obtain more quantitative data, a complementary approach using tandem mass tag labeling of peptides was performed. Here, we identified 31 sites in 29 proteins exhibiting at least 2-fold induction (Figure 6C and Supplemental Table 4). Importantly, the 14 proteins present in both data sets include the transcriptional corepressors SAFB and SAFB2 (Figure 6C and Supplemental Table 4), which function as repressors of immune regulators, including MHC-I genes (42). Intriguingly, the full repressive activity of SAFB and SAFB2 was reported to rely on its SUMOylation (42).

To first confirm SUMO2/3 modification of SAFB, we performed anti-SUMO2/3 IP followed by SAFB immunoblotting in U-2-OS cells that were either treated with control or SUMOi. In whole-cell lysates of control cells but not SUMOi-treated cells, a higher molecular anti-SAFB reactive band was detectable (Figure 6D). This SAFB-conjugate was highly enriched in SUMO2/3 IPs, but was completely absent in the samples treated with SUMOi, demonstrating that it corresponded to SUMOylated SAFB (Figure 6D). The presence of multiple SUMO-SAFB conjugates in the IPs is in line with the MS-based identification of multiple SUMOylation sites in SAFB (Figure 6D). To further validate that SUMOylation of SAFB is induced by MYC and SUMOi can also prevent this induction, anti-SUMO2/3 IP was done in control cells and cells expressing MYC in either the presence or the absence of SUMOi. Consistent with the MS data, the level of SAFB-SUMO2/3 conjugates was strongly elevated upon induction of MYC, and SUMOi fully compromised this effect (Figure 6E). SUMOylation of SAFB at lysine 294, which we identified as an MYC-induced SUMOylation site by MS, is critical for its full repressive activity (ref. 43 and Figure 6E), which strongly suggests that MYC-induced SUMOylation is mechanistically linked to MHC-I repression.

Of note, inhibition of SUMOylation also affected SAFB protein levels, whereas SAFB2 levels were not affected (Figure 6, F–H). Likewise, we could not detect any effect on the transcript abundance of SAFB (Supplemental Figure 7, B–D), and MYC protein expression correlated with SAFB protein but not with SAFB mRNA across a large panel of cancer cells lines from the CCLE (Supplemental Figure 7E). To investigate whether depletion of SAFB alone is sufficient to restore MHC-I expression, we performed shRNA-mediated depletion of SAFB in the OCI-Ly1 DLBCL cell line. Depletion of SAFB significantly increased MHC-I expression (Figure 6I). Moreover, we observed similar effects when we performed siRNA-mediated depletion of SAFB in U-2-OS cells (Supplemental Figure 7F), thus identifying SAFB as a critical regulator of MHC-I. Importantly, SAFB depletion compromised the effect of SUMOi treatment on MHC-I induction, demonstrating that SAFB and SUMO act in a common pathway on MHC-I regulation (Supplemental Figure 8A). Next, we explored whether the loss of SAFB protein can explain the observed transcriptional induction of the MHC-I/APM pathway after SUMOi treatment (Figure 1H). To this end, we analyzed the transcriptome of SAFB-depleted cells (42) and found significant induction of *HLA-A*, *HLA-B*, *B2M*, *TAP1*, *LMP2*, and *LMP7* after SAFB depletion (Supplemental Figure 7, G–I), which phenocopied the effects of SUMOi. Moreover, GSEA identified enrichment of the gene set “Class I MHC mediated Antigen Processing and Presentation,” underscoring the role of SAFB as repressor of the MHC-I/APM pathway and its regulators (Supplemental Figure 7J). Importantly, SAFB depletion did not affect SUMOi-driven induction of STAT1 expression or STAT1 phosphorylation in response to IFN-γ in OCI-Ly1 cells (Supplemental Figure 8, B and C).

In summary, we identified a previously unknown mechanism of SUMO-mediated basal repression of the MHC-I/APM pathway and SAFB as its SUMO-regulated transcriptional repressor.

### SUMOi drives a feed-forward loop amplifying the antitumor immune response.

**Figure 7:**
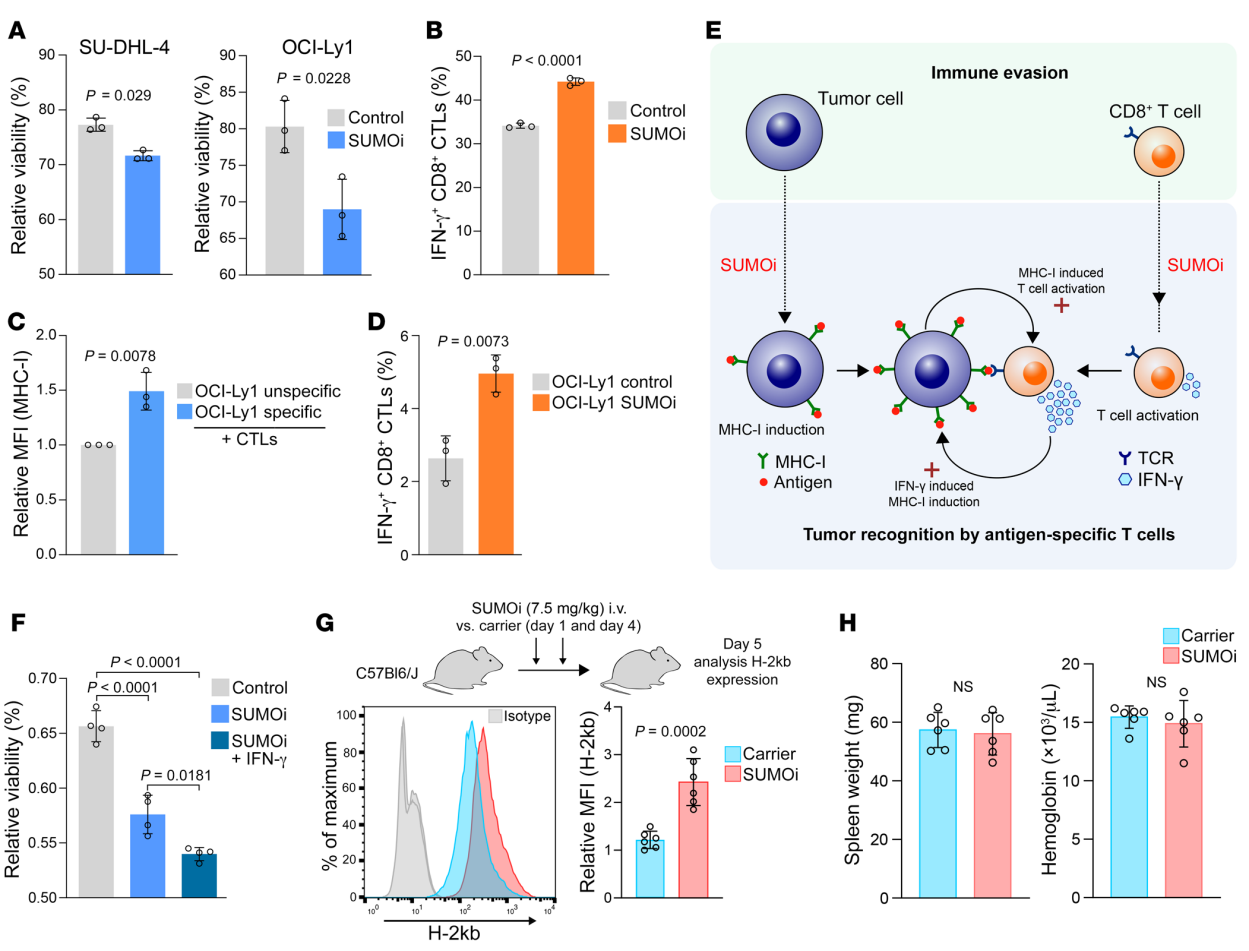
SUMOi drives a feed-forward loop amplifying the antitumor immune response. (**A**) Viability of OCI-Ly1 (40 nM SUMOi, 48 h) and SU-DHL4 (100 nM SUMOi, 48 h) cells (loaded with 2.5 μM peptide for 2 h) after coculturing with CTLs at effector/target ratio 5:1 for 5 h. DAPI staining and flow cytometry measurement (*n =* 3). *P* values determined by unpaired *t* test. (**B**) IFN-γ in CTLs treated with SUMOi (100 nM, 48 h) or control after coculturing for 16 hours with OCI-Ly1 cells (loaded with 2.5 M peptide for 2 h) at an effector/target ratio of 5:1 (*n =* 3). *P* value was determined by unpaired *t* test. (**C**) MHC-I expression on OCI-Ly1 cells loaded with a specific influenza or nonspecific control peptide (2.5 μM, 2 h) after coculturing for 12 hours with influenza-specific CTLs at an effector/target ratio of 2:1 (*n =* 3). *P* value was determined by unpaired *t* test. (**D**) IFN-γ expression in CTLs after coculturing for 16 hours with control or SUMOi-pretreated OCI-Ly1 cells (40 nM, 48 h, loaded with 0.02 μM peptide for 2 h) at an effector/target ratio of 5:1 (*n =* 3). *P* value was determined by unpaired *t* test. (**E**) SUMOi drives induction of the MHC-I/APM pathway in tumor cells and the activation of T cells. When both cell types are combined, SUMOi drives a feed-forward mechanism amplifying the antitumor immune response. (**F**) Viability of OCI-Ly1 cells (40 nM SUMOi, 48 h) incubated or not with IFN-γ (100 U/mL) for 24 hours (loaded with 2.5 μM peptide for 2 h) after coculturing for 5 hours with CTLs at an effector/target ratio of 5:1. DAPI staining and flow cytometric measurement (*n =* 4). *P* values were determined by ANOVA with Tukey’s post hoc test. (**G**) Experimental workflow and H-2Kb expression on B cells in WT mice treated with SUMOi (*n =* 6) or carrier (*n =* 6). *P* value was determined by unpaired *t* test. (**H**) Comparison of spleen weights and hemoglobin levels in WT mice treated with SUMOi (*n =* 6) or carrier (*n =* 6). *P* value was determined by unpaired *t* test. All data in the figure represent the mean ± SD.

The implementation of immunotherapies has revolutionized cancer therapies and clinical practice. Despite the striking success, often only subgroups of patients respond (32). CD8^+^ CTLs recognize target cells displaying specific antigens presented by the MHC-I/APM pathway and are the key effectors in patients undergoing ICB (26). The loss of the MHC-I/APM pathway and the IFN-γ/STAT1 axis as one of its regulatory mechanisms has been identified as a frequent cause of resistance to ICB (26, 32, 44). Thus, strategies to restore these pathways may enhance the efficacy of immunotherapies, and a recent study showed profound activating effects of SUMOi on immune cells and improved survival in preclinical models after treatment with SUMOi and ICB (45). To directly test the functional relevance of SUMOi-triggered MHC-I restoration on tumor cells with regard to CD8^+^ T cell killing of target cells, we took advantage of a coculture model in which the specific interaction between tumor cells and CTLs is mediated by influenza peptide. In fact, DLBCL cells with SUMOi-restored MHC-I surface levels were more sensitive to T cell killing than control cells, revealing that SUMOi-restored MHC-I expression had functional consequences for the antitumor immune response (Figure 7A). In a potential clinical application, both tumor cells and T cells would be subject to SUMOi. Therefore, we tested whether SUMOi treatment also affects the activation status of CD8^+^ T cells. SUMOi-treated CD8^+^ CTLs produced substantially higher levels of IFN-γ, indicating increased T cell activation (Figure 7B). This was further empowered by increased STAT1 phosphorylation (Supplemental Figure 9A) and by transcriptome profiling results indicating enrichment of type I and type II IFN signaling (Supplemental Figure 9, B–D). Moreover, cytokine production by activated T cells further augmented MHC-I expression on cancer cells (Figure 7C), which typically boosts the activation of CD8^+^ T cells and cytokine production. Accordingly, SUMOi-pretreated tumor cells cocultured with CD8^+^ T cells enhanced T cell activation, as indicated by increased IFN-γ production when compared with the coculture with control tumor cells (Figure 7D). Of note, knockout of *HLA-A* and consequently depletion of MHC-I diminished the increased sensitivity to T cell killing and the activation of CTLs (Supplemental Figure 10). Thus, the SUMOi-mediated induction of MHC-I and the SUMOi-induced activation of CD8^+^ T cells drive a feed-forward loop, which amplifies the effects of SUMOi observed in the separate systems (Figure 7E). To test whether this mechanism could indeed amplify the antitumor immune response, we pretreated DLBCL cells with SUMOi and IFN-γ to maximally induce the MHC-I/APM pathway. In line with the suggested mechanism, the combination of SUMOi and IFN-γ showed the highest susceptibility to CTL-induced cytolysis (Figure 7F).

Finally, to test the in vivo potential of SUMOi as inducer of MHC-I expression, we treated WT mice with SUMOi and analyzed MHC-I expression (Figure 7G). Despite the high MHC-I expression of murine WT B cells, SUMOi significantly enhanced MHC-I expression (Figure 7G), whereas we did not observe any signs of toxicity in SUMOi-treated mice (Figure 7H).

In summary, these data identified a SUMOi-driven feed-forward loop amplifying the antitumor immune response. Moreover, our findings revealed SUMOi as a potential therapeutic strategy to enhance the efficacy of cancer immunotherapies.

### SUMOi globally alters the immune landscape.

**Figure 8:**
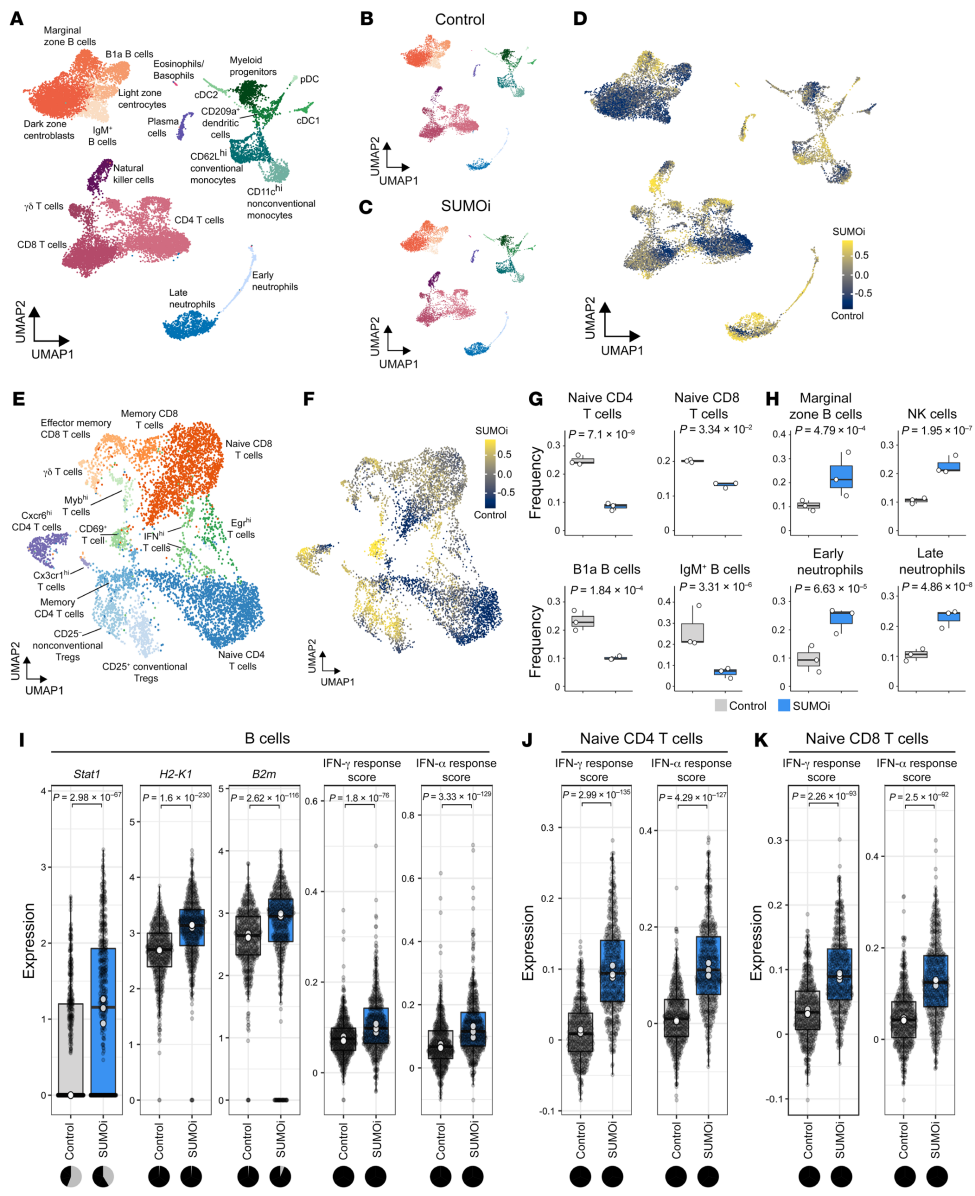
SUMOi globally alters the immune landscape. (**A**) UMAP visualization of spleen scRNA-Seq data from control and SUMOi-treated mice. (**B**) UMAP visualization of spleen scRNA-Seq data from control mice (*n =* 3). (**C**) UMAP visualization of spleen scRNA-Seq data from SUMOi-treated mice (*n =* 3). (**D**) Detection of differentially abundant cell populations in the spleens of control and SUMOi-treated mice using DA-Seq (71). Cells are colored by the DA-Seq measure. Yellow indicates greater abundance after SUMOi treatment; dark blue indicates greater abundance in the control. (**E**) The T cell populations identified in **A** were separated and reclustered. The UMAP visualization shows T cells for both conditions. (**F**) Detection of differentially abundant T cell populations in control and SUMOi-treated mice with DA-Seq. Cells are colored by the DA-Seq measure. Yellow indicates greater abundance after SUMOi treatment; dark blue indicates greater abundance in control mice. (**G** and **H**) Differential abundance testing on mouse-wise pseudo-bulks (white dots, *n* = 3). Bar plots indicate the respective subpopulation frequencies stratified by condition. The center line of the box plot is the median. The box extends from the 25th to 75th percentiles. The whisker length is from minimum to maximum. Significance was determined using a negative binomial generalized linear model. (**G**) Significantly more abundant cell populations were detected in control mice. (**H**) Significantly more abundant cell populations were detected in SUMOi-treated mice. (**I**–**K**) Differential expression analysis in B cells (**I**), naive CD4^+^ T cells (**J**), and naive CD8^+^ T cells (**K**) of the genes of interest (normalized expression) and IFN response scores (arbitrary expression). Gray dots represent individual cells. White dots indicate the median per mouse-wise pseudo-bulk. The back line indicates the median across all cells. Wilcoxon’s rank-sum test was applied to determine significance. The adjusted *P* values (Bonferroni’s correction) are shown. The pie charts indicate the number of cells with normalized counts equal to 0 (gray) and normalized counts greater than 0 (black) for the respective genes and condition.

Our previous investigations revealed multifaceted changes upon SUMOi in lymphoma cells and T cells. To systematically assess the effect of SUMOi in hematopoietic cells, we next performed cellular indexing of transcriptomes and epitopes by sequencing (CITE-Seq) (46). CITE-Seq enables the combined analyses of surface protein and transcriptome measurements to comprehensively measure cellular states and their alterations in a tissue system at the single-cell level (46). Spleen cells from control and SUMOi-treated mice (Figure 7G) were tagged with oligo-conjugated antibodies to distinguish different B cell and T cell subsets, followed by transcriptomic and surface-proteomic profiling on the 10x Genomics platform. In total, our data set comprised 18,361 high-quality cells (9878 control, 8483 SUMOi treated) with a median detection of 1636 genes and 3955 unique molecular identifiers (UMIs) per single cell. We identified all major cell populations or stages of differentiation physiologically prevalent in the spleen (Figure 8A). Overall, our analysis revealed a substantial remodeling of the immune system after SUMOi treatment in vivo, thus underscoring the broad effects and complexity of a SUMO-targeted intervention (Figure 8, A–D). Specifically, naive CD4^+^ and CD8^+^ T cells, as well as B cells, were less abundant (Figure 8, D–G), whereas the abundance of NK cells and early and late neutrophils was substantially higher in SUMOi-treated mice (Figure 8, D–H). Of note, type I and type II IFN signaling were activated in naive CD4^+^ and CD8^+^ T cells and the abundance of activated T cell subsets was increased, suggesting an antigen-independent T cell activation phenotype (Figure 8, E–G, J and K, and Supplemental Figure 11). Accordingly, we observed a significantly higher abundance of CD69 T cells in the spleen of SUMOi-treated mice (Figure 8, E and F), which is in line with our previous data that SUMOi activates tumor-infiltrating CD8^+^ T cells (Figure 4F). Of note, SUMO inhibition transcriptionally induced the APM of nonmalignant splenic B cells (e.g., expression of *H2-k1*, *B2m*) and resulted in a global induction of type I and type II IFN signaling, expanding our findings from BCL toward normal B cells (Figure 8I). Beyond this, these data revealed a striking complexity of SUMOi treatment on the immune landscape and suggest that SUMOi affects the immune response by several mechanisms.

## Discussion

Starting from a targeted approach for the discovery of SUMO-coordinated immune escape mechanisms in B cell non-Hodgkin lymphomas (B-NHLs), we here unraveled an evolutionary conserved mechanism with broad physiological relevance for cell biologists and immunologists and defined a potential strategy to enhance the efficacy of cancer immunotherapies.

Immune surveillance creates an effective barrier to tumorigenesis and tumor progression. Consequently, cancer cells have acquired distinct escape strategies to overcome these limitations. CD8^+^ CTLs are key players of the antitumor adaptive immune response. Typically, they recognize target cells via antigens processed and presented by the MHC-I APM. This in turn drives T cell activation and target cell killing (26). Genetic loss and transcriptional silencing of MHC-I APM genes is an established cause of primary and acquired resistance to cancer therapies employing the host immune system like ICB (25, 26). Therefore, strategies to reactivate the MHC-I/APM pathway are considered promising strategies to enhance the efficacies of ICB (47) and currently tested cellular therapies, e.g., TCR T cell therapy.

We identified a conserved function of activated SUMOylation in protecting tumor cells against immune destruction, which is further reinforced by a recent genome-wide screening for MHC-I regulators in DLBCL that also identified SUMO2 as a candidate negative MHC-I regulator (48). We demonstrated that activated SUMOylation mediated the coordinated repression of critical MHC-I APM components, such as the immunoproteasome, the TAP transporters, and genes encoding the MHC-I complex. Thereby, we expanded the current understanding of SUMO’s function in the cellular stress response by uncovering SUMO as coordinator of physiological MHC-I expression hijacked into a tumor-intrinsic immune escape mechanism. Our study revealed that activation of the SUMOylation machinery conveyed a survival benefit to cancer cells in a hostile environment. So far, mainly genetic immune escape mechanisms have been identified. However, the growing body of research implicating nongenetic mechanisms for immune evasion strategies is pointing toward an essential role of posttranslational and epigenetic mechanisms in shaping the immunogenicity of tumor cells (26, 49). Expanding this concept, we here showed that activated SUMOylation drove immune escape in cancer in a nongenetic manner. Moreover, we suggest that inhibition of SUMOylation may be a valid therapeutic strategy to enhance the efficacy of cancer immunotherapies in a broad spectrum of solid and hematological cancers by activating MHC-I antigen presentation. This is of particular clinical interest considering that hyperactivated SUMOylation has been identified as a key feature in many different cancer entities, and several pivotal oncogenes activate the SUMOylation pathway (1, 21, 50). Of note, a recent study showed profound activating effects of SUMOi on immune cells via type I IFN signaling and that the combination of SUMOi with ICB improved survival in preclinical models (45). With our studies, we expand the understanding of SUMOi as a rational combinatorial treatment with ICB by establishing a significant contribution of a SUMOi-induced tumor-intrinsic mechanism. This provides critical information on the MHC-I APM as a potential predictive biomarker for this treatment strategy. Our data could thereby inform clinical study results and in the future directly inform patient cohorts for clinical design. Of note, we also showed experimentally that inhibition of SUMOylation was a powerful inducer of MHC-I APM in various cancer entities and thus provide a mechanistic framework and targeting strategy for a highly conserved feature of cancers of virtually all tissues.

Previous studies have linked the activity of the SUMOi TAK-981 to type I IFN signaling and showed that SUMOi could lead to activation of immune cells in mice (45, 51, 52). SUMOylation has an established function in limiting the hyperreactivity of cells in response to type II IFN signaling (40). Here, we demonstrated a function of SUMOylation in transcriptional repression of STAT1. Accordingly, SUMOi induced STAT1 transcript and protein expression, thus priming tumor cells for response to type II IFN. We also observed similar effects for STAT2, which might explain the activating effects on type I IFN signaling (53), which is in line with previous studies (45). In line with this finding, we have highlighted the role of activated SUMOylation as a barrier to cytokine-mediated transcriptional activation of the MHC-I APM in cancer and showed that SUMOylation is heavily involved in type II IFN signaling. This is of particular interest considering that SUMOi drives not only activation and IFN-γ secretion of CTLs, but also amplifies the IFN-γ–induced restoration of the tumor-intrinsic MHC-I suppression and thereby reconstitutes immune surveillance. Notably, TAK-981 is currently part of various clinical trials (ClinicalTrials.gov NCT03648372, NCT04074330, NCT04381650), highlighting the relevance of our findings for future clinical applications.

We and others have linked the oncogene MYC to activation of SUMOylation (21, 54). Notably, MYC was correlated with suppression of the MHC-I APM in studies 30 years ago (29). We have now experimentally addressed this observation and showed that activation of MYC indeed suppressed basal expression of the MHC-I APM and functionally correlated this with enhanced immune evasion. Importantly, we showed that the MYC effect was dependent on SUMOylation and fully reversible by pharmacological SUMOi. Moreover, we would like to emphasize that the regulation of MYC by SUMOi is an area worthy of further investigation.

To uncover the SUMO-mediated mechanisms of MYC-induced suppression of MHC-I, we performed an unbiased MS-based analysis and identified the transcriptional repressor SAFB. Previous studies showed that SAFB is a negative transcriptional regulator of MHC-I APM (42). SAFB is an established SUMO substrate and SUMOylation is crucial for its repressive activity (43). Of note, we showed that MYC directly induced SUMOylation of SAFB, which links MYC activity to repression of MHC-I. In addition to this model, we here showed that SUMOylation might affect the stability of SAFB, but not SAFB2, and that inhibition of SUMOylation caused a dose-dependent drop in SAFB protein levels. We showed that this mechanism is not only valid in lymphoma, but also in nonhematopoietic cancers, thus identifying a highly conserved mechanism. However, the exact mode of SAFB stabilization and whether it is directly caused by SUMO modification of SAFB needs to be addressed in further studies.

We and others have identified inhibition of SUMOylation as therapeutic strategy in a broad spectrum of cancers (21, 54, 55). Consequently, several synthetic inhibitors of the SUMO pathway have been developed, most prominently TAK-981. TAK-981 inhibits the activation of SUMO by the dimeric E1 enzyme. Mechanistically, SUMOi causes G2/M arrest and mitotic failure in tumor cells, which can be explained by the broad implication of SUMOylation in mitosis (54, 56). In this study, we report that SUMOi enhanced the immunogenicity of tumor cells by transcriptionally inducing MHC-I APM. Together with our finding of SUMOi-driven activation of CD8^+^ T cells, we here propose a SUMOi-dependent feed-forward mechanism enhancing antitumor immunity. We have thus established the concept of a double-targeting strategy by SUMOi in cancer. From a clinical perspective, highly immunogenic tumor cells are frequently eliminated by the immune system. This process, referred to as immunoediting, favors the growth of less immunogenic cancer cells and is an established cause of resistance to cancer immunotherapies. Therefore, augmenting the immunogenicity of tumor cells is of particular therapeutic interest, and we suggest inhibition of SUMOylation as a therapeutic strategy to enhance the efficacy of immunotherapies in a broad spectrum of cancers.

## Methods

### Chemicals.

TAK-981 was either purchased from MedChemExpress or provided by Millennium Pharmaceuticals, Inc., a wholly owned subsidiary of Takeda Pharmaceutical Company Limited. TAK-981 doses and treatment durations are indicated in the figure legends. Recombinant human IFN-γ was purchased from PeproTech. For IFN-γ–induced MHC-I induction, cells were treated with 100 U/mL for 24 hours. Doxycycline hyclate (D9891) was obtained from Sigma-Aldrich. A concentration of 1 μg/mL was used to induce MYC expression on U-2-OS cells with a doxycycline-inducible MYC construct.

### Viral infection and cell culture.

Human DLBCL cell lines were kept in RPMI-1640 (SU-DHL-4/5/6, Toledo, RIVA, HBL-1. and TMD8) or IMDM (OCI-Ly1) medium supplemented with 10% to 20% FCS, 1% penicillin streptomycin (P/S), and 2 mM L-glutamine. U-2-OS cells with inducible MYC expression have been described (31) and were provided by M. Eilers (University of Würzburg, Würzburg, Germany). U-2-OS cells were grown in DMEM with 10% FCS and 1% P/S. P493-6 cells were provided by D. Eick (Helmholtz Zentrum, Munich, Germany) and propagated in RPMI with 10% FCS, 1% L-glutamine, and 1% P/S; suppression of MYC was induced by 0.1 μg/mL doxycycline for 48 hours. B16-OVA cells were grown in DMEM with 10% FSC and 1% P/S. Kelly cells were cultured in RPMI-1640 supplemented with 10% FCS, 1% P/S, and 1 mM sodium pyruvate. MCF-7 cells were cultured in IMDM supplemented with 20% FCS. H1299 cells were cultured in RPMI supplemented with 10% FCS and 1% P/S. CT26 cells were purchased from ATCC and were modified to overexpress murine epithelial cell adhesion molecule (EpCAM) as previously described (57). Cells were cultured in RPMI containing 10% FBS, 2 mM L-glutamine, and 100 U/mL penicillin and 100 μg/mL streptomycin. For the generation of lentiviral particles, HEK293T cells were cotransfected with the indicated lentiviral plasmids and viral packaging plasmids (Lipofectamine 2000, Invitrogen). For shRNA knockdown, specific shRNA constructs targeting human *SAFB* were ordered from Sigma MISSION (SAFB: TRCN0000022101). Virus supernatants were collected 48 hours after transfection and used to transduce the indicated cell lines in the presence of 1 μg/mL polybrene (Sigma-Aldrich). Suspension cells were transduced using spin-transduction at 400*g* for 1 hour at 32°C.

### Transfection of siRNAs.

siRNA transfections were performed with Lipofectamine RNAiMAX transfection reagent (Thermo Fisher Scientific). For a reverse transfection, the siRNAs were combined with Opti-MEM and mixed, and then Lipofectamine RNAiMAX was added. After incubation at room temperature for 20 minutes, this mix was added to the cell suspension. The mixture was plated evenly and incubated for 72 hours at 37°C.

### Flow cytometry.

Cells were washed in HF2 buffer (ddH2O, 2% FCS, 1% P/S, 1% HEPES, 10% HBSS) and stained on ice for 30 minutes in HF2 (a list of all antibodies is provided in the supplemental material). After washing in HF2, cells were either resuspended in HF2 containing DAPI for FACS analysis or fixed with BD Biosciences Cytofix/Cytoperm for intracellular staining. Data were acquired on Beckman Coulter CytoFLEX S.

### Immunoblot analysis.

Protein extracts were prepared by solving cell pellets in lysis buffer (150 mM NaCl, 1% NP-40 Oder IGEPAL, 0.5% sodium deoxycholate, 0.1% SDS, 50 mM Tris) supplemented with NaF, PMSF, and NaVO_4_ followed by sonification. Protein lysates were fractioned on SDS PAGE gels, transferred to PVDF transfer membrane (Thermo Fisher Scientific), and incubated with specific antibodies. A list of all antibodies is provided in the supplemental material.

### SUMO IP.

SUMO2/3-specific 8A2 hybridoma clone A11 (provided by Frauke Melchior, Zentrum für Molekulare Biologie der Universität Heidelberg [ZMBH], University of Heidelberg, DKFZ – ZMBH Alliance, Heidelberg, Germany) was harvested as described by Barysch et al. (58). Subsequent Western blot analysis was performed as described (58). MS-based SUMOylome analysis was done as described by Hendriks et al. (41).

### MS-based proteome analysis.

A detailed description of the methods is provided in Supplemental Methods.

### CRISPR/Cas9-based gene editing.

For depletion of B2M and HLA-A in the human OCI-Ly1 DLBCL cell line, a major part of exon 1 of the B2M open reading frame and a fragment ranging from exon 3 to exon 4 of the HLA-A open reading frame was removed by CRISPR/Cas9 gene editing. To this end, 150,000 OCI-Ly1 cells were transfected with 500 ng of each of the sgRNAs (sgRNA sequences are listed in Supplemental Methods) and 1 μg Cas9 protein (PNA Bio) with a Neon Transfection System (Thermo Fisher Scientific/Invitrogen) (parameters: 1600 V; 10 ms; 3 pulses). The cleavage efficacy was tested 24 hours after transfection with the Terra PCR Direct mix and primers flanking the target exons. Cells were then separated into single cells by serial dilution. Cell clones were screened for efficient gene editing, and selected clones were analyzed for B2M protein expression by immunoblot analysis or MHC-I expression by flow cytometry.

### Reverse transcription qPCR.

RNA was isolated using the RNeasy Mini Kit (QIAGEN). cDNA was generated using the MMLV HP Reverse Transcriptase (Lucigen) or in a 1-step protocol with the Luna universal One-Step RT-qPCR Kit (NEB). qPCR was performed using a PikoReal 96 cycler (Thermo Fisher Scientific) or a StepOnePlus cycler and the Maxima SYBR Green/ROX qPCR Master Mix (2×) (Thermo Fisher Scientific) and analyzed using the ΔΔCt method with control samples set as 1. All primer sequences are provided in the supplemental material.

### RNA-Seq and processing of gene expression data.

Cells and RNA isolation were prepared as described for reverse transcription qPCR. RNA quality was assessed with Agilent RNA 6000 Pico Kit according to the manufacturer’s instructions in the Agilent Bioanalyzer 2100. RNA concentration was determined with a Nanodrop spectrophotometer. Library preparation and paired-end sequencing were performed by Novogene on a HiSeq2500 (Illumina) with a sequencing depth of more than 25 M reads/sample. The resulting Fastq files were mapped to the human reference genome hg38 as described (59). Reads were estimated for each transcript using the transcript sequences from the human reference hg38 and the Salmon software (60). Counts were normalized and differential gene expression was analyzed by DeSeq2 (61). Additional gene expression data were retrieved from NCBI’s GEO (GSE7897, GSE32219, GSE4475, and GSE15548). Affymetrix Array data were either normalized and extracted using the Expression Console software as described (62) or normalized counts supplied by the authors were log_2_ transformed before downstream analysis. Normalized count tables were subsequently used for GSEA, using the Kolmogorov-Smirnov test and Hallmark and Reactome Signatures of the Molecular Signature Database (63) implemented in GeneTrail 3.0 (64). Differential gene expression analysis between conditions was carried out using DeSeq2 (61). Selected gene expression results were illustrated in heatmaps using ClustVis (65).

### Generation of peptide-specific CTLs.

Production of influenza-peptide-specific CTLs was adapted from Woelfl et al. (66). Heparinized peripheral blood samples were obtained from HLA-A2–positive healthy individuals. PBMCs were isolated by Ficoll density gradient centrifugation. CD8 separation was done by MACS MicroBeads isolation. The CD8^–^ fraction was kept in 6-well plates for 2 hours to allow adherence to plastic. Adherent cells were cultured in presence of GM-CSF (800 U/mL) and IL-4 (1000 U/mL) for 48 hours to obtain DCs. DC maturation was induced by adding IL-4 (1000 U/mL), LPS (10 ng/mL), and IFN-γ (100 U/mL) overnight. Mature DCs were then loaded with influenza peptide (2.5 μg/mL) for 2 hours at 37°C before coculturing with CD8^+^ cells (ratio DC/CD8^+^ = 1:10) in the presence of IL-7 and IL-15 (5 ng/mL) for 10 days. Medium and cytokines were replaced every 2 to 3 days.

### Cytolysis assay.

Cancer cells were pretreated as previously described with SUMOi or DMSO control for 48 hours and incubated with or without IFN-γ for 24 hours before use. Cells were then loaded with influenza peptide or control peptide (2.5 μg/mL, otherwise indicated in the figure legends) for 2 hours at 37°C. After washing, cells were cocultured with influenza-specific CTLs at indicated effector/target ratios.

### Peptide pulsing assay.

Murine 483 cells were pretreated with 10 nM SUMOi or DMSO control for 72 hours. After washing, cells were pulsed with 100 ng/mL SIINFEKL (Ova peptide) at 37°C for 2 hours to allow binding to cell-surface MHC-I (H-2Kb).

### Animal experiments.

BALB/c (4–6 weeks old, female) and C57Bl6/J (4 to 6 weeks old, female) mice were purchased from Charles River Laboratories (C57Bl6/J: 027; BALB/c: 028). BALB/c mice were injected (s.c.) with 2 million CT26-EpCAM cells into the right flank. When the tumor size reached 2 × 2 mm, mice were randomized and i.v. treated with 2 doses of 7.5 mg/kg SUMOi or appropriate vehicle control on days 1 and 4. Mice were euthanized 24 hours after receiving the second dose. Flow cytometric analysis of the tumors was carried out as previously described (67). C57Bl6/J mice were treated with SUMOi following the same scheme. *E-myc* [B6.Cg-Tg(IghMyc)22Bri/J] mice were obtained from The Jackson Laboratory (stock no. 002728). Male and female mice were examined twice a week and euthanized as soon as lymph nodes were well-palpable (5 mm diameter) or any of the approved thresholds were reached.

### Bioinformatic analysis.

To determine the activity of the antigen processing and presentation core machinery (*LMP2*, *LMP7*, *TAP1*, *TAP2*, *B2M*, *HLA-A*, *HLA-B*, *HLA-C*), we extracted expression data of cell lines listed in the CCLE (https://depmap.org/portal/) and performed ssGSEA. Each dot represents the normalized enrichment score (NES) for individual cancer cell lines; the black line indicates the median. Cell lines were classified according to cancer type, and cancer types were sorted according to their median NES. Profiling of tumor-infiltrating immune cells was performed using CIBERSORT according to Chen et al. (68).

### Data availability.

The transcriptome data generated in this study have been deposited at the EBI European Nucleotide Archive under accession number PRJEB49824. The single-cell RNA-Seq data generated in this study have been deposited in NCBI’s GEO (GSE193359). The MS proteomics data generated in this study have been deposited in the ProteomeXchange Consortium via the PRIDE partner repository (69, 70) with the data set identifiers PXD030506 and 10.6019/PXD030506.

### Statistics.

Statistical analyses were performed using GraphPad Prism. The error bars shown in the figures represent the SD, unless specified otherwise. A *P* value lower than 0.05 was generally considered significant, and all exact *P* values and tests are indicated in the figures.

### Study approval.

All animal experiments were performed in accordance with local authorities (Regierung von Oberbayern, Munich, Germany, and LAGeSo Berlin, Germany).

## Author contributions

UMD, SM, MW, MS, and UK conceived and designed the study. UMD, MB, SY, CG, LZ, PWH, AG, HK, KI, DV, FG, ER, KW, JD, VI, BB, FB, S Habringer, JL, BC, CW, SK, S Haas, ABB, MW, and MS acquired and/or analyzed and interpreted data. UMD, SM, MS, MW, and UK drafted the manuscript. All authors revised the manuscript for important intellectual content and approved the final version submitted for publication.

## Supplementary Material

Supplemental data

Supplemental table 1

Supplemental table 2

Supplemental table 3

Supplemental table 4

## Version 1 05/02/2022

Electronic publication
