# Supplementary material for: Activated SUMOylation restricts MHC class I antigen presentation to confer immune evasion in cancer
Source: J Clin Invest. 2022 May 2;132(9):e152383. doi: 10.1172/JCI152383 (PMC9057585; doi:10.1172/JCI152383)
Supplement: Supplemental data [file jci-132-152383-s134.pdf]

Activated SUMOylation restricts MHC class I antigen presentation to confer immune evasion in cancer

Demel et al.

MS-based proteome analysis

Desalting, TMT labelling and high pH fractionation. For proteome analysis cells were lysed, reduced and alkylated in SDS-lysis Buffer (2% SDS, 150mM NaCl, 50mM Tris pH 8.5, protease inhibitor tablet, 5mM TCEP, 40mM CAA). After boiling and sonicating, samples were precipitated using MeOH/Chloroform precipitation. The pellet was then resolved in Urea digestion buffer (8M urea, 50mM TrisHCl, pH 8.2). Digestion of proteins was performed in 1M urea, 50mM TrisHCl, pH 8.2, using Trypsin and Lys-C in 1:100 enzymes to protein ratio. Peptides were subsequently desalted using C18 Sep-Pak-Cartridges. 10μg of digested peptides was dried and reconstituted in 10 μl 200 mM EPPS pH 8.2; 20 % ACN. TMT labelling was performed using TMT reagents supplied by ThermoFisherScientific(1). Peptides were purified through another round of Stage Tipping or high pH fractionation. Therefore, peptides were fractionated using High pH Reversed phase fractionation kit (ThermoFisher Scientific). The latter was used according to manufacturer's instructions.

Liquid Chromatography and Mass Spectrometry (MS). Peptides were analyzed on a Q Exactive HF/Orbitrap Fusion Lumos Tribrid Mass Spectrometer (SUMO-IP/Proteome) or coupled to an easy nLC 1200 (ThermoFisher Scientific) using a 20 cm/30cm long, 75 μm ID fused-silica column packed in house with 1.9 μm C18 particles (Reprosil pur, Dr. Maisch), and kept at 50 °C using an integrated column oven (Sonation). Peptides were eluted by different non-linear gradient from 13–32/5-40 % acetonitrile over 70 min (IPs) or 4–30 % acetonitrile over 120 min (proteomes) and directly sprayed into the mass-spectrometer equipped with a nanoFlex ion source (ThermoFisher Scientific). The mass spectrometer was operated in a data-dependent mode. Full-scan MS spectra of samples were acquired covering a mass range of 400–1600/350-1400 m/z using 3E6/4E5 as AGC target with a resolution of 60.000 at 200 m/z and a maximum injection time of 60 ms. The 15/20 most intense ions were fragmented by Higher-energy collisional dissociation (HCD) applying a normalized collision energy of 25/35 %. Resolution for MS/MS spectra was set to 60.000/50.000 at 200 m/z, AGC target to 1E5, maximal injection time to 120 ms/30 ms. For Proteome Analysis repeated sequencing of already acquired precursors was limited by setting a dynamic exclusion of 60 s and 7 ppm, and advanced peak determination was deactivated.

Raw data analysis. All RAW files were analyzed using MaxQuant software versions 1.5.8.3-1.6.10.43(1, 2). Unless stated otherwise, default MaxQuant settings were used. SUMOylome analysis was performed as described by Hendriks et al. 2018(3). Proteome analysis was performed using default MaxQuant settings, beside specification of TMT correction factors and enzyme cleavage specificity.

Pathway enrichment analysis of proteins. Pathway enrichment analysis was performed by GeneTrail2(4) (using Reactome database) and was assessed using an over-representation

analysis. Corresponding *P-values* are FDR-adjusted per database using the method of Benjamini and Yekutieli with a significance level of 0.05. For enrichment analysis, proteins which were significantly enriched after SUMOi treatment in SU-DHL-4 and/or OCI-Ly1 cells were chosen. Results obtained with Reactome have been visualized with GraphPad.

Generation of STRING network. The freely available STRING software (version 11.0) was used to generate the STRING network. For network analysis, proteins which were significantly enriched after SUMOi treatment in SU-DHL-4 and/or OCI-Ly1 cells were chosen. For all analyses, we set the parameters to highest confidence and used a kmeans clustering with 7 clusters. Experiments and databases were enabled. Non-connected proteins were excluded from the visualized interaction network.

Fluorescence-activated cell sorting, ADT labeling and cell hashing of mouse spleens
Spleens were dissected and stored in RPMI-1640 containing 10% FCS. Single cell suspensions were obtained by pressing individual spleens through a 40 µm mesh and subsequent washing with 10 ml FACS buffer (DPBS, 5 % FCS, 0.5mM EDTA). After centrifugation for 5 min at 400g, red blood cells were lysed by resuspending cells in 1ml of ACK lysis buffer (Lonza) and incubation for 3 min at RT. Cells were washed with FACS buffer and centrifuged for 5 min at 400g. Subsequently, cells from individual spleens were resuspended in FACS buffer containing H2-Kb-biotin antibody and incubated for 15 min on ice. Cells were washed and centrifuged for 5 min at 400g and individually resuspended in FACS buffer containing NK1.1 BV711, B220 PE-Cy7, CD3 APC, CD11b APC-Cy7 antibodies as well oligo-conjugated streptavidin PE and oligo-conjugated CD4, CD8, CD44, CD62L, CD25, IgM, IgD and TotalSeq anti mouse hashtag antibodies. Cells were incubated on ice for 25 min, washed with 1 ml FACS buffer and centrifuged for 5 min at 400g. This step was repeated for overall three washes. For cell sorting, cells were resuspended in FACS buffer and Caspase 3/7 and DAPI were added prior to cell sorting for apoptotic and dead cell exclusion. $3x10^3$ B cells, $3x10^3$ T/NK cells and $3x10^3$ remaining cells were sorted per mouse and overall cells from 3 mice from every treatment group (control/SUMOi) were pooled and loaded separately in 2 reactions of the 10x Chromium chip according to manufacturer’s instructions. After that, single-cell libraries were generated according to the protocol Chromium Next GEM Single Cell 3' v3.1 with Feature Barcoding technology for Cell Surface Protein.

<u>scRNA-seq analysis</u>

Preprocessing and quality assessment of scRNA-seq data

Demultiplexed fastq files were processed using Cell Ranger (version 6.0.1, 10x Genomics). The reads were aligned to the *Mus musculus* reference genome GRCm38 (mm10). The generated UMI (unique molecular identifier) count matrices were imported into R (version 4.1.0) and further processed using the Seurat package (version 4.0.5). Only cells with >=200

detected genes and <10% mitochondrial reads were retained for downstream analyses. To remove outliers, upper filtering thresholds for the number of genes and UMIs were set at >=6,000 and >=40,000 respectively. Furthermore, only genes detected in >= 3 cells were kept. Using the expression information from hashtag oligos (HTO), samples were demultiplexed and only singlets were included for subsequent analyses. HTO demultiplexing was done using Seurat's HTODemux function with default parameters. Sample quality was additionally assessed by performing a clustering per condition (control/SUMOi). We found that the three samples were distributed equally among all clusters within the respective treatment group, indicating no hashing bias. Using Seurat's FindMarker function, signature genes for each cluster were determined. Cell populations with co-expression of marker genes for distinct cell types were labeled as doublet clusters and removed prior to data integration.

Data integration, clustering, and cell type annotation

After quality assessment and removal of low-quality cells, samples were integrated to account for batch effects. Data integration was conducted using Seurat's standard integration workflow. In brief, raw RNA counts were log-normalized to account for differences in library size and the top 2,000 variable genes were identified. Then, integration features were selected and integration anchors determined using CCA (canonical correlation analysis). After scaling, a principal component analysis (PCA) was run on the integrated object and the top 30 principal components (PCs) were used for SNN (shared nearest neighbor) graph construction, clustering (based on Louvain algorithm) and UMAP visualization. For cell type identification, Seurat's FindMarker function (with default parameters) was applied to obtain signature genes for each cluster. Together with knowledge-derived gene lists, cell populations were manually annotated (see Main Figure 8A). After cluster assignment, CD4 T cells, CD8 T cells and γδ T cells were separated and reclustered according to the workflow described above using the top 20 PCs (see Figure 8E).

Differential abundance analysis

To detect differentially abundant cell populations DA-seq was applied (5). The analysis was conducted according to the tutorial (on https://klugerlab.github.io/DAseq/articles/tutorial.html) with values for *k* ranging from 20 to 500 for both the global and the T cell object (see Figure 8D and Figure 8F), respectively. Although DA-seq can identify differentially abundant subpopulations without requiring clustering information, it does not return any statistics. Therefore, to quantify abundance levels, we additionally performed differential abundance testing using mouse-wise pseudobulks (see Boxplots in Figure 8G and 8H). For this analysis

the global dataset was used in which T cells annotations were replaced with the more detailed labels identified in Figure 8E (separate T cell clustering). Initially, cell counts for each sample (mouse) and population were determined. The cell type proportions (=frequency) for each cluster and sample were calculated by dividing the respective number of cells by the total number of cells within that cluster. For statistics, design and contrast matrices were constructed using the model.matrix (stats package version 4.1.0) and makeContrasts function (limma package version 3.48.1). Finally, a Negative Binomial Generalized Linear Model was fitted using the glmFit function (edgeR package version 3.34.0) and likelihood ratio tests were conducted using glmLRT (edgeR package).

Differential expression analysis

Genes of interest and interferon response scores were subject of differential expression testing. Hallmark interferon α and γ response gene lists were downloaded from https://www.gsea-msigdb.org, imported into R, and converted to mouse homologs. IFN-α and IFN-γ response scores were generated using Seurat's AddModuleScore function. To assess whether genes of interest and interferon response scores were differentially expressed across conditions (control/SUMOi), a two-sided Wilcoxon rank sum test was applied. For this, we used Seurat's FindMarker function with test.use = "wilcox". As input, the log-normalized expression values (genes of interest) or arbitrary expression levels (interferon scores) were provided.

Antibodies

Epitope	Reactivity	Flurochrome	Company	Catalogue number
MHC-I (HLA-A, B, C)	human	APC	Biolegend	311410
MHC-II (HLA-DR, DP, DQ)	human	APC	Biolegend	361713
IgG2a,k Isotype	mouse	APC	Biolegend	400220
IFNy	human	APC	BD Bioscience	562017
CD8	human	PECy7	Biolegend	344711
CD20	human	PE	BeckmannCoulter	IMI1451
CD45	human	APC	eBioscience	17-9459-42
CD47	human	APC	Biolegend	323123
CD54	human	PE	Biolegend	322707
CD58	human	-	BD Bioscience	555919
CD80	human	PECy7	BD Bioscience	561135
CD86	human	PB	Biolegend	305423
CD 274 (PD-L1)	human	APC	Biolegend	329707
CD261 (DR4, TRAIL_R1)	human	APC	Biolegend	307207
CD95 (FAS)	human	-	Sigma-Aldrich	05-201
CD178 (FAS-L)	human	BV-421	Biolegend	306411

CD119 (IFN-y-Ra chain)	human	PE	Biolegend	308703
Annexin V	human	APC	Biolegend	640941
MHC-I (H-2kB)	mouse	APC	ThermoFisher	17-5958-82
H-2Kb bound to SIINFEKL	mouse	APC	Biolegend	400220
MHC-I (H-2kD)	mouse	APC	Biolegend	114714
CD3	mouse	PE	Biolegend	100308
CD4	mouse	BV605, AF700	Biolegend	100451, 100430
CD8	mouse	BV786	Biolegend	100750
CD45	mouse	PE-Cy7	Biolegend	103114
CD25	mouse	PE-Dazzle	Biolegend	101920
CD69	mouse	BV510	Biolegend	104532
Lag3	mouse	PerCP Cy5.5	Biolegend	125212
PD1	mouse	BV421, BV605	Biolegend	135221, 135220
EpCAM	mouse	FITC	Biolegend	118208
NK-1.1	mouse	BV711	Biolegend	108745
CD3	mouse	APC	Biolegend	100235
B220	mouse	PE-Cy7	Biolegend	103221
CD11b	mouse	APC-Cy7	Biolegend	301341
DAPI	all	-	Biolegend	422801
Caspase-3/7	all	-	ThermoFisher	C10723

CITE-Seq Reagents

Epitope	Reactivity	Flurochrome	Company	Catalogue number
CD4	mouse	TotalSeq-B001	Biolegend	100573
CD8a	mouse	TotalSeq-B002	Biolegend	100783
CD44	mouse	TotalSeq-B0073	Biolegend	103071
CD62L	mouse	TotaSeq-B0012	Biolegend	104465
CD25	mouse	TotalSeq-B0097	Biolegend	102067
IgM	mouse	TotalSeq-B0450	Biolegend	406545
IgD	mouse	TotalSeq-B0571	Biolegend	405751
H-2kB	mouse	Biotin	Biolegend	116503
Streptavidin	mouse	PE TotalSeq-B0952	Biolegend	405287
Hashtag 1	mouse	TotalSeq-B0301	Biolegend	155831
Hashtag 2	mouse	TotalSeq-B0302	Biolegend	155833
Hashtag 3	mouse	TotalSeq-B0303	Biolegend	155835

SingleCell-Seq Reagents

Name	Company	Catalogue number

Chromium Next GEM Single Cell 3’ GEM, Library & Gel Bead Kit v3.1, 4 rxns	10x Genomics	PN-1000128
Chromium™ Single Cell 3' Feature Barcode Library Kit, 16 rxns	10x Genomics	PN-1000079

Westernblot Antibodies

Target	Company	Article number
STAT1	Cell signaling	14994S
pSTAT1 (Y701)	Cell signaling	9167S
SAFB	Abcam	ab 187650
SAFB2	Abcam	ab 104220
MYC	Cell signaling	9402S
SUMO1	Cell signaling	4930S
SUMO2/3	Cell signaling	4971S
B2M	Cell signaling	12851S
eIF4E	Cell signaling	2067T
β-Actin	Sigma-Aldrich	A1978
β-Tubulin	DSHB	E7

qPCR Primers

Gene Human	Sequence
HLA-A	Fw: GGCCCTGACCCAGACCTG<br>Rv: GCACGAACTGCGTGTCGTC
HLA-B	Fw: CATCGTGGGCATTGTTGCTG<br>Rv: ACGCAGCCTGAGAGTAGC
HLA-C	Fw: CTGGCCCTGACCGAGACCTG<br>Rv: CGCTTGTACTTCTGTGTCTCC
B2M	Fw: TGACTTTGTCACAGCCCAAG<br>Rv: AGCAAGCAAGCAGAATTTGG
TAP1	Fw: TCAGGGCTTTCGTACAGGAG<br>Rv: TCCGGAAACCGTGTGTACTT
TAP2	Fw: ACTGCATCCTGGATCTCCC<br>Rv: TCGACTCACCCTCCTTTCTC
LMP2 (PSMB8)	Fw: TCAAACACTCGGTTCACCAC<br>Rv: GGAGAAGTCCACACCGGG
LMP7 (PSMB9)	Fw: CATGGGCCATCTCAATCTG<br>Rv: TCTCCAGAGCTCGCTTTACC
SAFB	Fw: CCCGAAGATGACTCGGATACAA<br>Rv: TGTAGAAGAGAGTCCACTAACCCAGA
SAFB2	Fw: GATCTCAAGAACCTTTTCAGCAAGTAT<br>Rv: TCGACATGGTGACGAATCCA
STAT1	Fw: CAGCTTGACTCAAAATTCCTGGA<br>Rv: TGAAGATTACGCTTGCTTTTCCT
SUMO1	Fw: TTCAACTGAGGACTTGGGGG<br>Rv: TGGAACACCCTGTCTTTGAC
SUMO2	Fw: GCCGACGAAAAGCCCAAGG<br>Rv: TGACAATCCCTGTCGTTCACAA
GAPDH	Fw: GGTATCGTGGAAGGACTCATGAC<br>Rv: ATGCCAGTGAGCTTCCCGTTCAG
β-Actin	Fw: GTCGAGTCGCGTCCACC<br>Rv: GTCATCCATGGCGAACTGGT

CRISPR/Cas9 sgRNA sequences

sgRNA Target	sgRNA Sequence
HLAA_Ex3_g2 fw	taatacgactcactataGGGACCGCGACGACACTGATgttttagagctagaaatagc
HLAA_Ex4_g3 rv	taatacgactcactataGGCACTCACCGGCCTCGCTCgttttagagctagaaatagc
B2M_Ex1_g2 fw	taatacgactcactataGGGCACGCGTTTAATATAAGgttttagagctagaaatagc
B2M_Ex1_g3 rv	taatacgactcactataGGGGTAGGAGAGACTCACGCgttttagagctagaaatagc

siRNA sequences

siRNA Target	siRNA Sequence
Control	CGUACGCGGAAUACUUCGATT
SUMO1 #1	GAGAAUUGCUGAUAAU
SUMO1 #2	GGACAGGAUAGCAGUGAGATdT
SUMO2 #1	AGAACAACGAUCAUAUUAATT
SUMO2 #2	GUCAAUGAGGCAGAUCAGATdT

1. Thompson A, Schafer J, Kuhn K, Kienle S, Schwarz J, Schmidt G, et al. Tandem mass tags: a novel quantification strategy for comparative analysis of complex protein mixtures by MS/MS. *Anal Chem.* 2003;75(8):1895-904.
2. Cox J, and Mann M. MaxQuant enables high peptide identification rates, individualized p.p.b.-range mass accuracies and proteome-wide protein quantification. *Nat Biotechnol.* 2008;26(12):1367-72.
3. Hendriks IA, Lyon D, Su D, Skotte NH, Daniel JA, Jensen LJ, et al. Site-specific characterization of endogenous SUMOylation across species and organs. *Nat Commun.* 2018;9(1):2456.
4. Stockel D, Kehl T, Trampert P, Schneider L, Backes C, Ludwig N, et al. Multi-omics enrichment analysis using the GeneTrail2 web service. *Bioinformatics.* 2016;32(10):1502-8.
5. Zhao J, Jaffe A, Li H, Lindenbaum O, Sefik E, Jackson R, et al. Detection of differentially abundant cell subpopulations in scRNA-seq data. *Proc Natl Acad Sci U S A.* 2021;118(22).

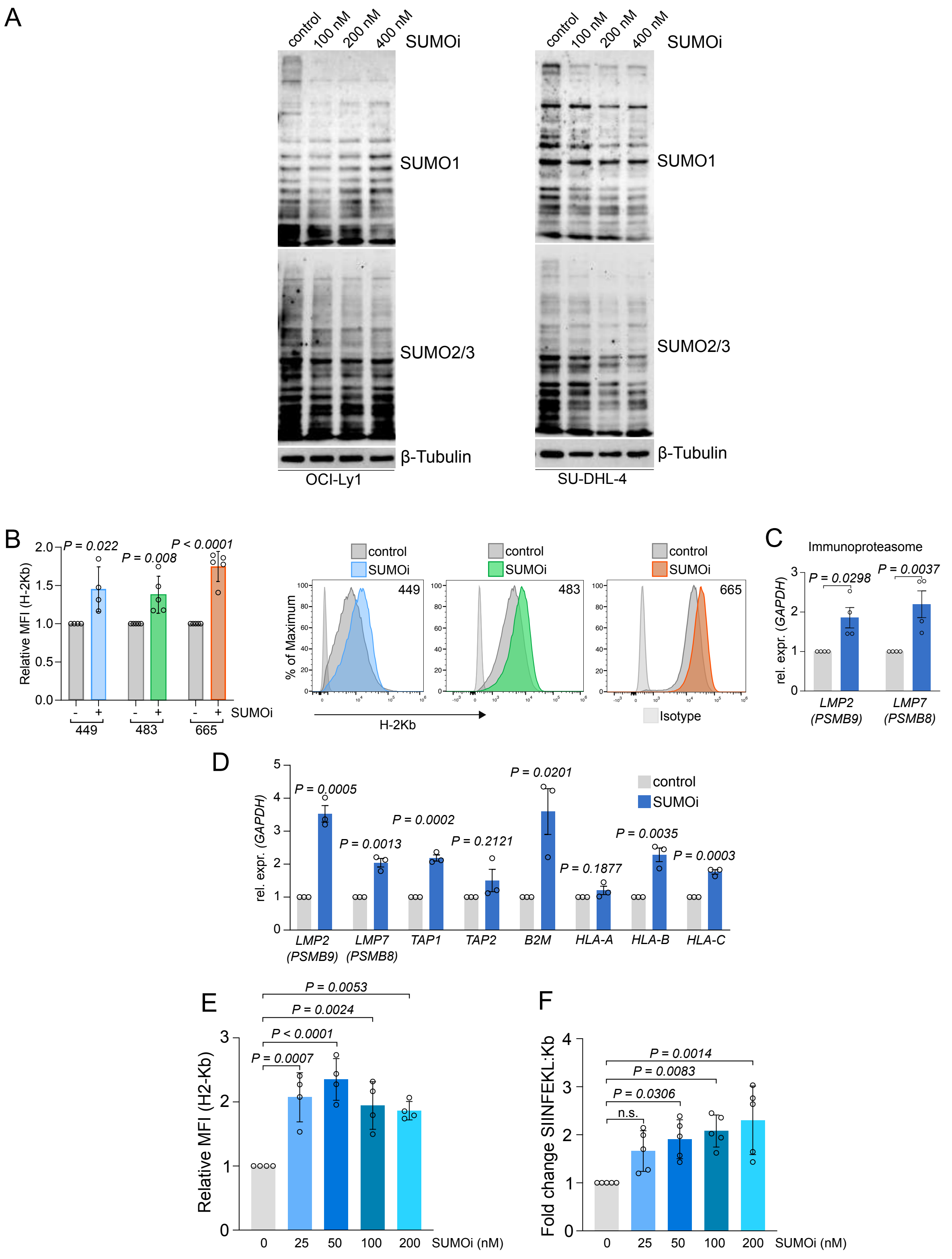

Supplementary Figure S1. (A) Immunoblot analysis of OCI-Ly1 and SU-DHL-4 cells treated with control or the indicated SUMOi concentrations for 72 h. **(B)** MHC-I expression of murine Eμ-myc lymphoma cell lines treated with SUMOi (449: 50nM, 483: 10nM, 665: 50nM) or control for 72h (n=4). Data represent mean +/- SD. P-values were determined by unpaired t-test. **(C)** mRNA expression analysis of indicated MHC-I APM genes in SU-DHL-4 cells treated with SUMOi (100nM, 72h) or control (n=4). Data represent mean +/- SD. P-values were determined by unpaired t-test. **(D)** mRNA expression analysis of indicated MHC-I APM genes in OCI-Ly1 cells treated with SUMOi (40nM, 72h) or control (n=3). Data represent mean +/- SD. P-values were determined by unpaired t-test. **(E)** MHC-I expression of murine B16-OVA cell line treated with SUMOi or control for 72h (n=4). Data represent mean +/- SD. P-values were determined by ANOVA; Tukey`s post hoc test. **(F)** Flow cytometry analysis of SIINFEKL:Kb of murine B16-OVA cell line treated with SUMOi or control (72h) (n=5). Data represent mean +/- SD. P-values were determined by ANOVA; Tukey`s post hoc test.

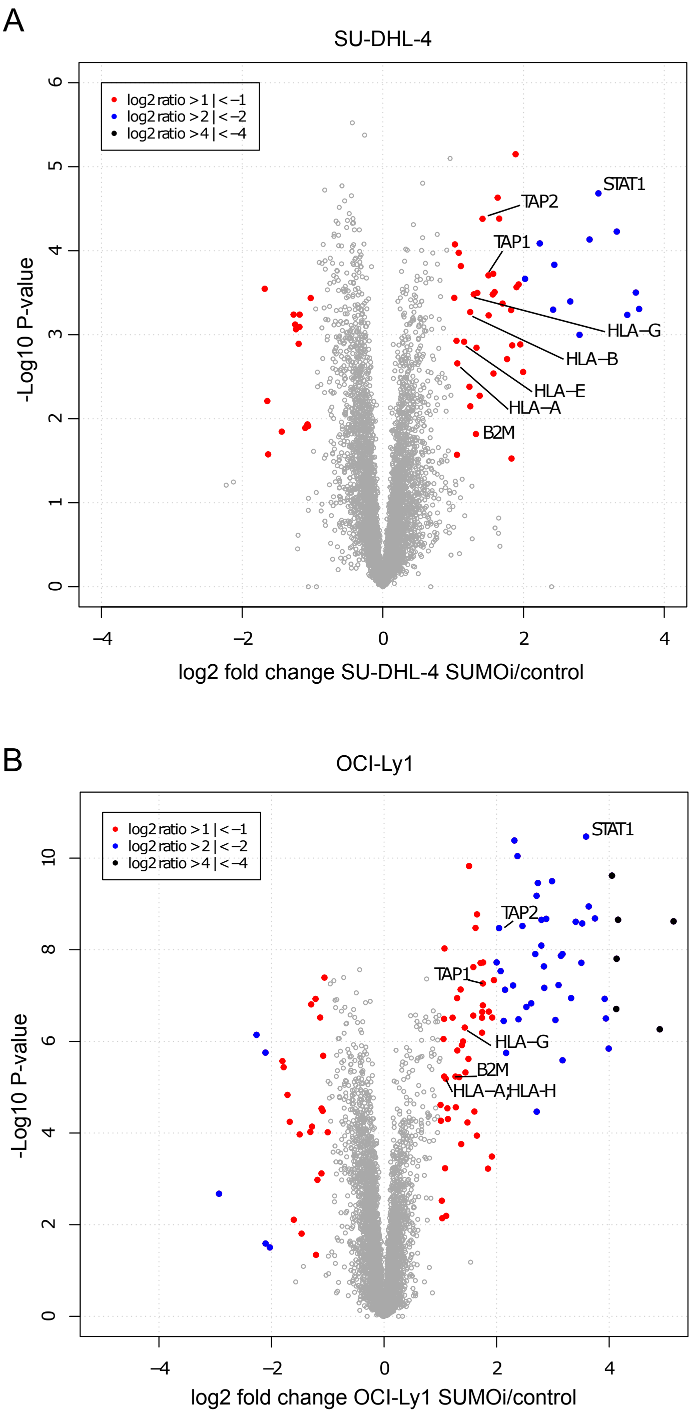

Supplementary Figure S2. (A, B) Volcano plot summarizing the results of quantitative MS analysis from the SU-DHL-4 and OCI-Ly1 DLBCL cell lines after SUMOi treatment (400 nM, 48 h). Proteins considered as significantly enriched are color-coded (cut-offs are indicated in the figure). The experiment was performed in triplicates.

A

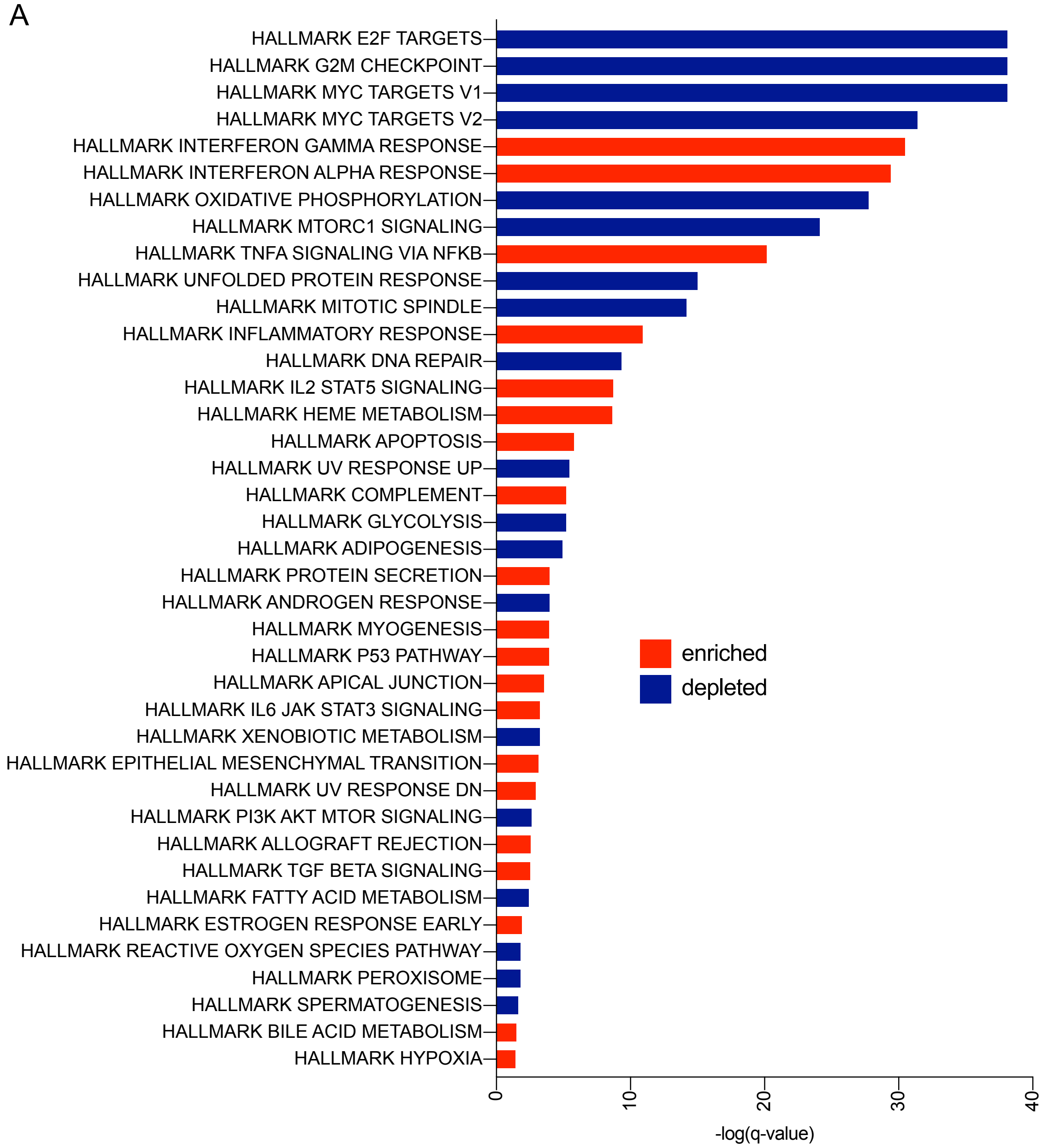

B

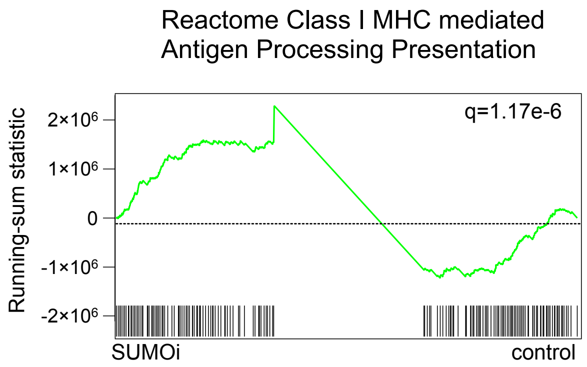

Supplementary Figure S3. (A) Summary of GSEA of expression data derived from transcriptome profiling SU-DHL-4 cells treated with control or SUMOi (400 nM, 48 h) with the indicated gene sets. P-value determined by Kolmogorov-Smirnov test. **(B)** GSEA of SU-DHL-4 cells treated with control or SUMOi (400 nM, 48 h) with the indicated gene set.

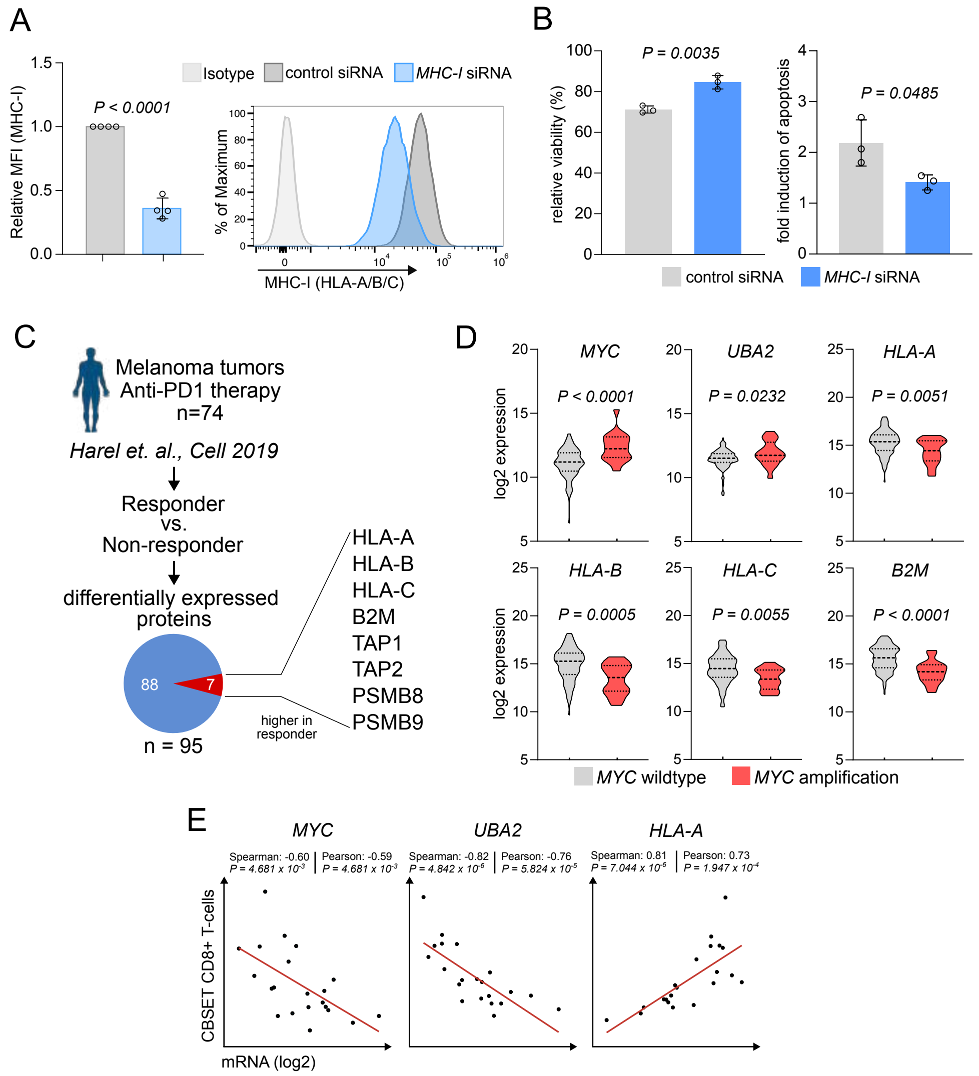

Supplementary Figure S4. **(A)** MHC-I expression of U-2-OS cells after transfection with either control or HLA-A siRNA (72 h, n=4). P-value was determined by unpaired t-test. **(B)** Flow cytometry analysis of cell death and apoptosis of U-2-OS cells after transfection with either control or HLA-A siRNA and incubation with CTLs at effector:target ratio 5:1 for 4.5 h. Viability determined by DAPI and AnnexinV staining (n=3). P-value was determined by unpaired t-test. **(C)** Graphical depiction of differentially expressed proteins of the MHC-I APM pathway identified in a study describing the proteome of human melanoma tumors following anti-PD1 therapy. The expression of all proteins was higher in the responder group. **(D)** Analysis of mRNA expression (log2 expression) of the indicated genes in primary human melanoma tumor (n=142) samples (data extracted from www.cbioportal.org). Groups were classified based on the MYC copy number status (TCGA). P-value was determined by unpaired t-test. **(E)** Correlation of mRNA expression (log2) of the indicated genes with CD8 T-cell infiltration estimated with CBSET algorithm (data extracted from www.cbioportal.org).

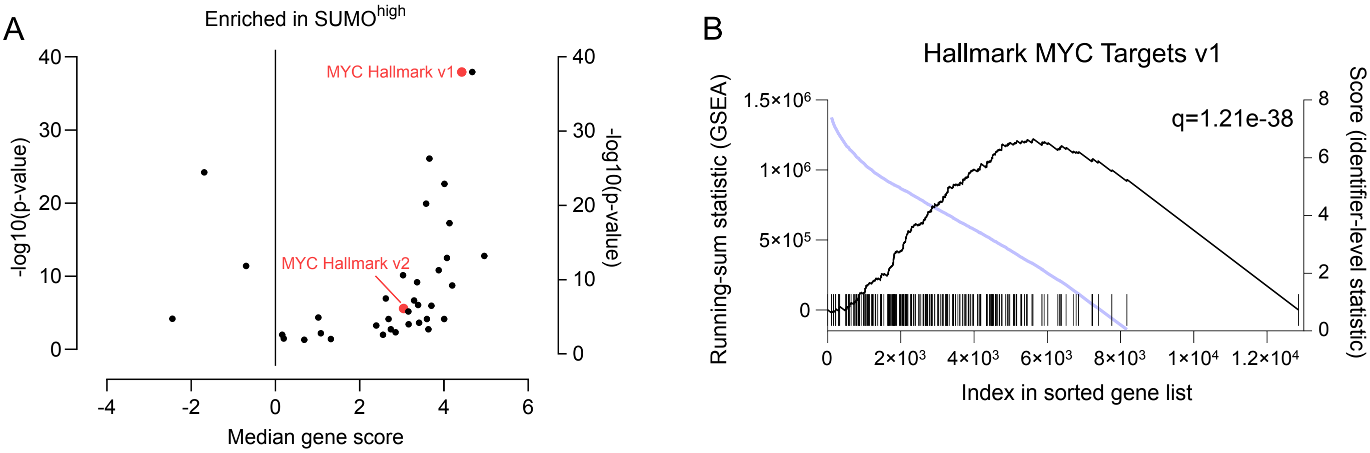

Supplementary Figure S5. (A) Summary of GSEA of SUMOhigh and SUMOlow DLBCL patient populations from Figure4A. Selected gene sets are highlighted. **(B)** GSEA of SUMOhigh and SUMOlow DLBCL patient populations from Figure4A with indicated gene set.

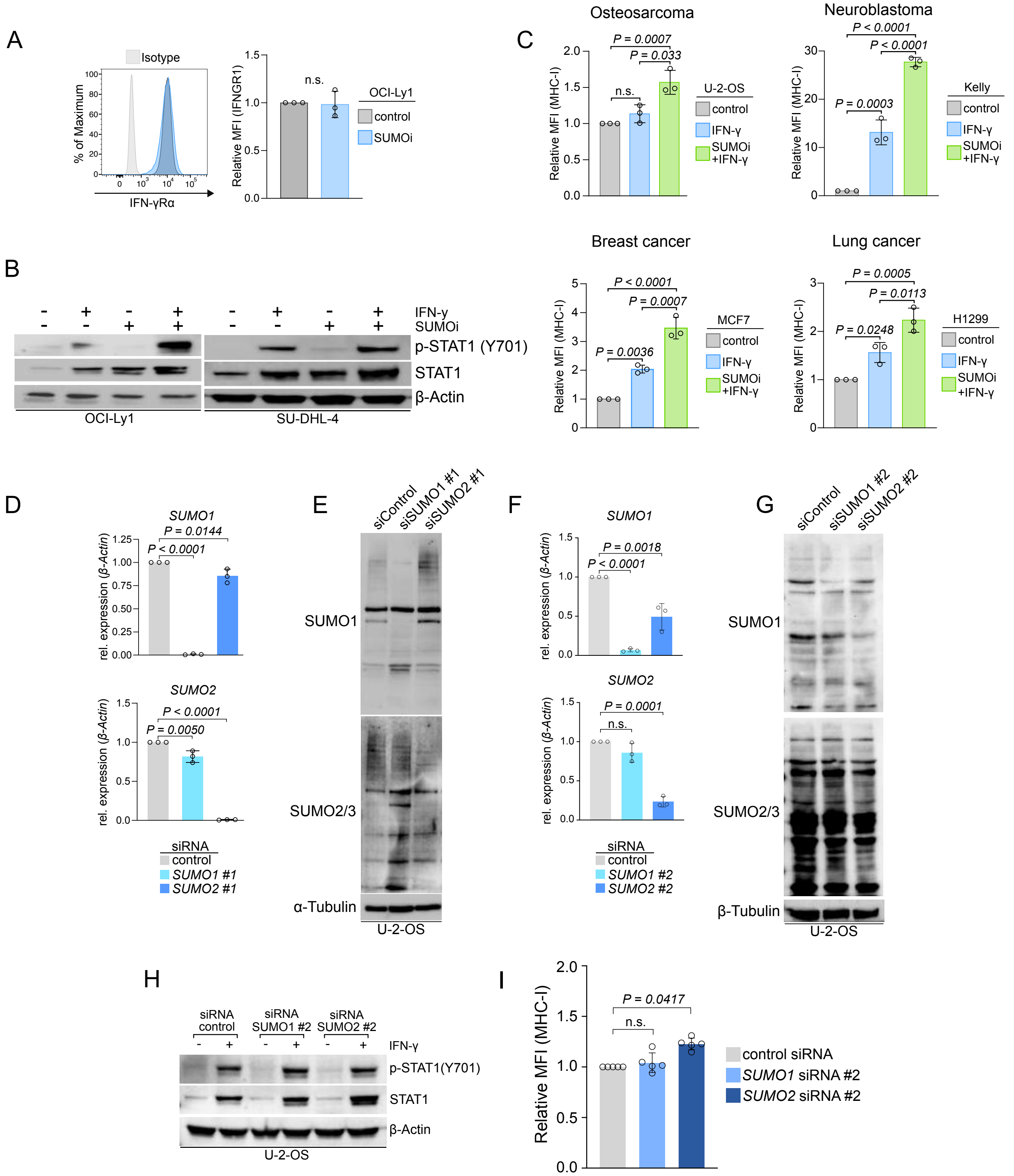

Supplementary Figure S6. (A) IFN-γRα expression of OCI-Ly1 cells treated with 40 nM SUMOi or control for 72 h (n=3). Data represent mean +/- SD. P-value was determined by unpaired t-test. **(B)** Immunoblot analysis of OCI-Ly1 (40 nM) and SU-DHL-4 (100 nM) cells treated with SUMOi or control for 72 h. Indicated samples were incubated with IFN-γ 100U/ml for 24h before harvest. **(C)** MHC-I expression following incubation with IFN-γ 100U/ml for 24 h on indicated cell lines pretreated +/- SUMOi (concentrations indicated in Figure 5G) (n=3). Data represent mean +/- SD. P-values were determined by ANOVA; Tukey`s post hoc test. **(D, F)** mRNA expression analysis of *SUMO1* and *SUMO2* of U-2 OS cells after transfection with two independent specific SUMO1, SUMO2 siRNAs or a control siRNA (72 h). **(E, G)** Immunoblot analysis of U-2 OS cells after transfection with two independent specific SUMO1, SUMO2 siRNAs or a control siRNA (72 h). **(H)** Immunoblot analysis of U-2-OS cells after transfection with specific SUMO1, SUMO2 or control siRNA (72 h) and treatment +/- IFN-γ 100 U/ml for 24 h. **(I)** MHC-I expression of U-2-OS cells after transfection with specific SUMO1, SUMO2 or control siRNA (72 h, n=3). Data represent mean +/- SD. P-values were determined by ANOVA; Tukey`s post hoc test.

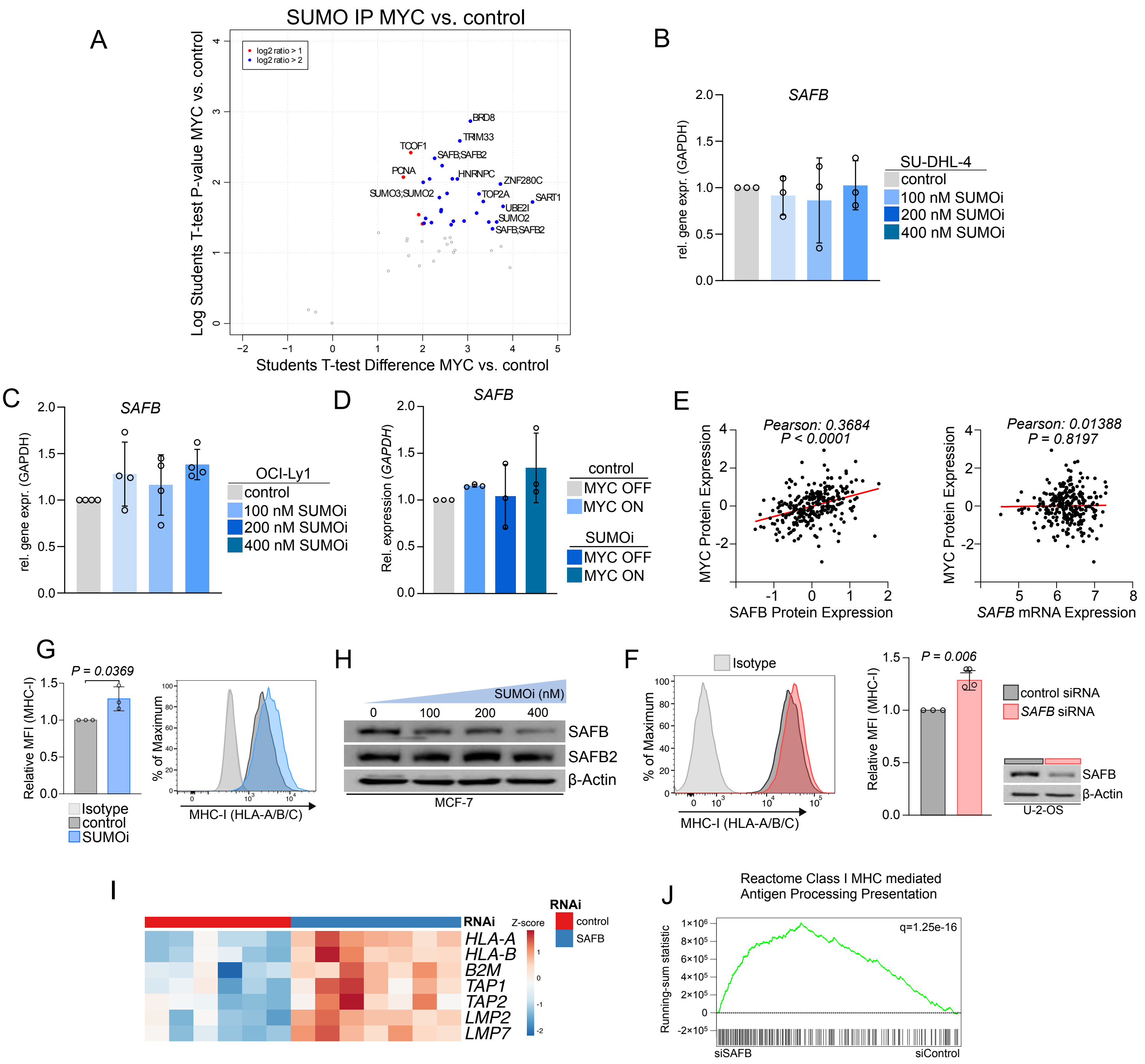

Supplementary Figure S7. (A) Volcano plot summarizing the results of quantitative MS analysis from the experiment described in Figure 6B. Hits considered as significantly enriched are color-coded (cut-offs are indicated in the figure). The experiment was performed in triplicates. **(B)** *SAFB* mRNA expression in SU-DHL-4 cells treated with increasing concentrations of SUMOi or control for 72 h. Data represent mean +/- SD. P-values were determined by ANOVA; Tukey`s post hoc test. **(C)** *SAFB* mRNA expression in OCI-Ly1 cells treated with increasing concentrations of SUMOi or control for 72 h. Data represent mean +/- SD. P-values were determined by ANOVA; Tukey`s post hoc test. **(D)** *SAFB* mRNA expression in U-2-OS cells treated with either 100 nM SUMOi or control for 72 h and 48 h of MYC induction. Data represent mean +/- SD. P-values were determined by ANOVA; Tukey`s post hoc test. **(E)** Correlation of MYC protein expression with either SAFB protein (left panel, n=277) or *SAFB* mRNA (right panel, n=272) expression across cell lines listed in the CCLE (data extracted from (https://depmap.org/portal/). **(F)** MHC-I expression of U-2-OS cells after transfection with specific SAFB siRNA or control siRNA (72h) (n=3). Data represent mean +/- SD. P-value was determined by unpaired t-test. Immunoblot analysis of the respective U-2-OS cells. **(G)** MHC-I expression of MCF-7 cells treated with 100 nM SUMOi or control for 72 h (n=3). Data represent mean +/- SD. P-value was determined by unpaired t-test. **(H)** Immunoblot analysis of MCF7 cells treated with indicated concentrations of SUMOi (0, 100, 200, 400nM) or control for 72h. **(I)** Expression of the indicated genes in MCF-7 cells following transfection with SAFB or control siRNA in the GSE15548 dataset. **(J)** GSEA of expression data derived from the GSE15548 dataset with the indicated gene set.

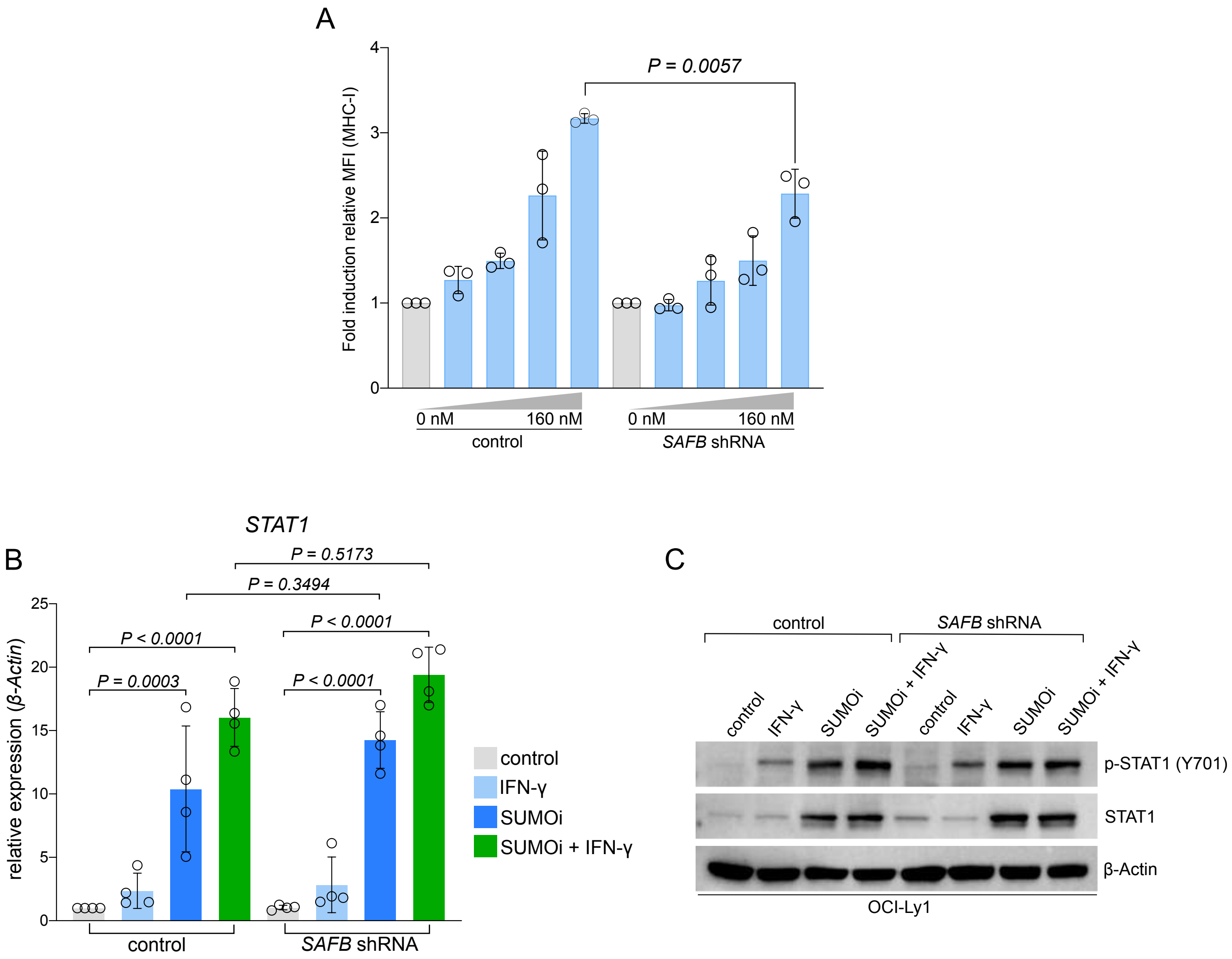

Supplementary Figure S8. (A) Fold induction of relative MHC-I expression of SAFB-depleted and control OCI-Ly1 cell lines from Figure 6I following control or SUMOi (20 nM, 40 nM, 80 nM, 160 nM) treatment. ***(B)*** *STAT1* mRNA expression analysis of SAFB-depleted and control OCI-Ly1 cell lines from Figure 6I following control or SUMOi (40 nM, 72 h) treatment and IFN-γ 100 U/ml for 1 h when indicated. Data represent mean +/- SD. P-values were determined by ANOVA; Tukey`s post hoc test. ***(C)*** Immunoblot analysis of SAFB-depleted and control OCI-Ly1 cell lines from Figure 6I following control or SUMOi (40 nM, 72 h) treatment and IFN-γ 100 U/ml for 1 h when indicated.

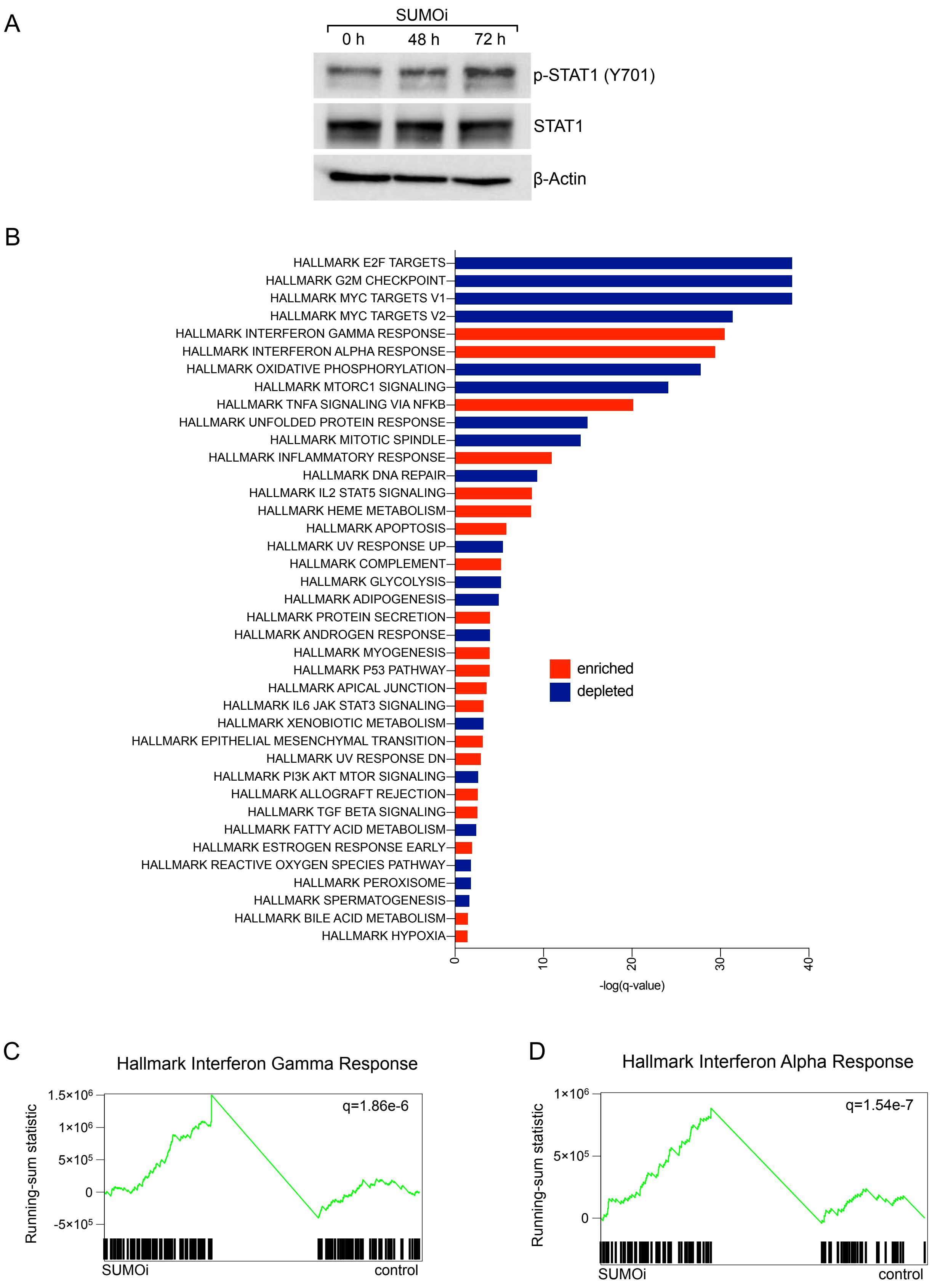

Supplementary Figure S9. (A) Immunoblot analysis of CTLs following SUMOi (100 nM) treatment for the indicated timepoints. **(B)** Summary of GSEA of expression data derived from transcriptome profiling of CTLs treated with control or SUMOi (100 nM, 72 h) with the indicated gene sets. P-value determined by Kolmogorov-Smirnov test. **(C, D)** GSEA of CTLs treated with control or SUMOi (100 nM, 72 h) with the indicated gene sets.

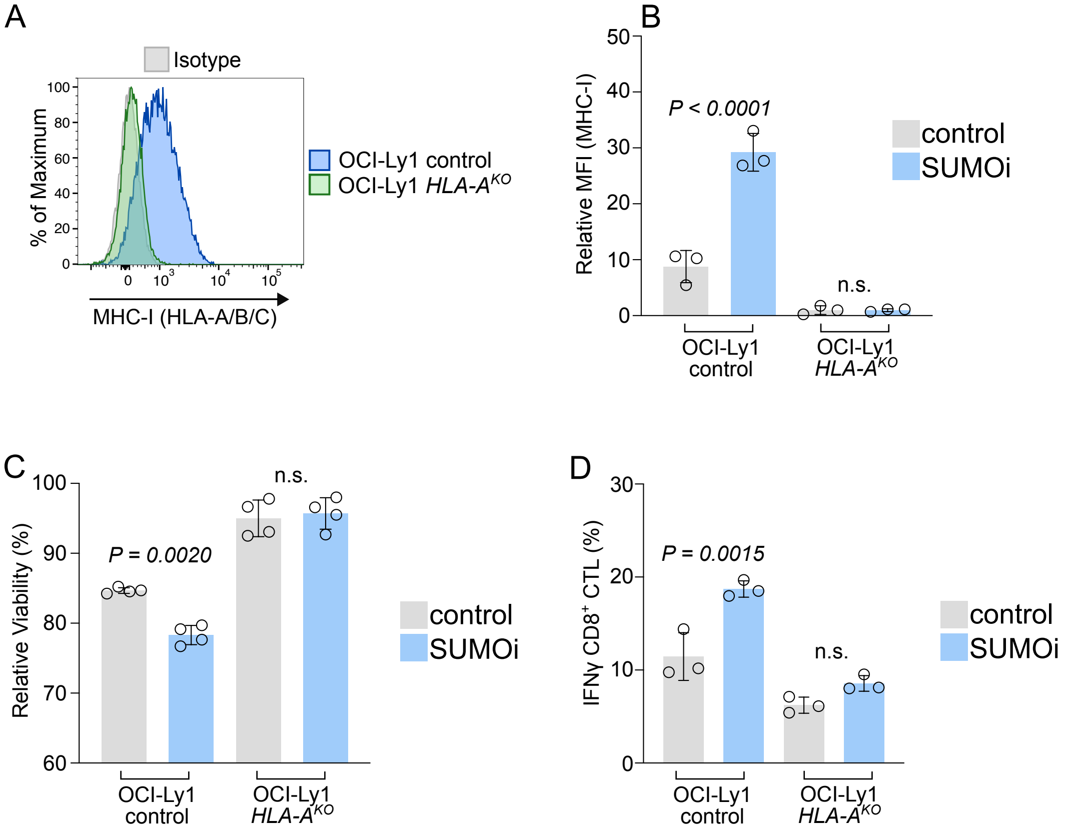

Supplementary Figure S10. (A) MHC-I expression of OCI-Ly1 control and *HLA-A*^{KO} cell lines. **(B)** MHC-I expression of OCI-Ly1 control and *HLA-A*^{KO} cell lines following control or SUMOi (40 nM) treatment for 72 h. **(C)** Viability of OCI-Ly1 (40 nM SUMOi, 48 h) cells (loaded with 2.5 µM peptide for 2 h) following co-culturing with CTLs at effector:target ratio 5:1 for 5 h. DAPI staining and flow cytometry measurement (n=4). Data represent mean +/- SD. P-values were determined by ANOVA; Tukey`s post hoc test. **(D)** IFN-γ in CTLs following co-culturing with control or SUMOi-pretreated OCI-Ly1 cells (40 nM, 48 h, loaded with 0.2 µM peptide for 2 h) at effector:target ratio 5:1 for 16 h (n=3). Data represent mean +/- SD. P-values were determined by ANOVA; Tukey`s post hoc test.

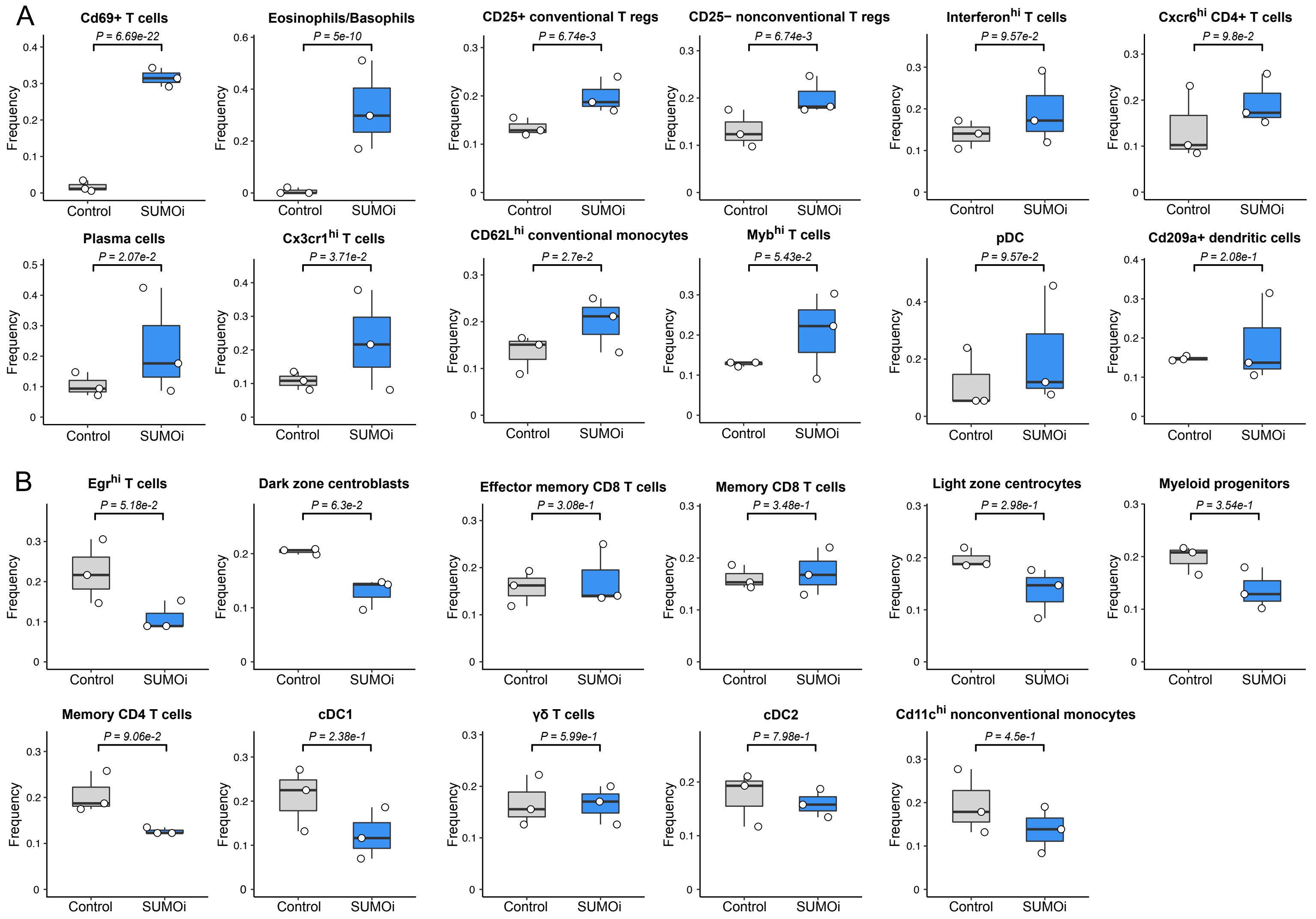

Supplementary Figure S11. (A, B) Differential abundance testing on mouse-wise pseudo bulks (white dots, n=3). The bar plots indicate the respective subpopulation frequencies stratified by condition. The center line of the box plot is the median. The box extends from the 25th to 75th percentiles. Whisker length is from minimum to maximum. Significance was determined by fitting a Negative Binomial Generalized Linear Model. **(A)** More abundant cell populations in SUMOi mice. **(B)** More abundant cell populations in control treated mice.
